# Design, Docking, Synthesis, and Biological Evaluation of Pyrazolone Derivatives as Potential Dual-Action Antimicrobial and Antiepileptic Agents

**DOI:** 10.3390/ph19020193

**Published:** 2026-01-23

**Authors:** Yousef Al-ebini, Manojmouli Chandramouli, Naga Prashant Koppuravuri, Thoppalada Yunus Pasha, Mohamed Rahamathulla, Salwa Eltawaty, Kamal Y. Thajudeen, Mohammed Muqtader Ahmed, Thippeswamy Boreddy Shivanandappa

**Affiliations:** 1 Department of Cosmetic Science, Faculty of Allied Medical Sciences, Al-Ahliyya Amman University, Amman 19328, Jordan; y.alebini@ammanu.edu; 2 Department of Pharmaceutical Chemistry, Faculty of Pharmacy, Sri Adichunchanagiri College of Pharmacy, Adichunchanagiri University, B. G. Nagara 571448, Karnataka, India; manojmouli@accp.co.in (M.C.);; 3 Department of Pharmaceutics, College of Pharmacy, King Khalid University, Abha 62223, Saudi Arabia; 4 Department of Biomedical Science, Faculty of Pharmacy, Omar Al-Mukhtar University, Albayda P.O. Box 991, Libya; 5 Department of Pharmacognosy, College of Pharmacy, King Khalid University, Abha 61421, Saudi Arabia; kthajudeen@kku.edu.sa; 6 Department of Pharmaceutics, College of Pharmacy, Prince Sattam Bin Abdul Aziz University, Al Kharj 11942, Saudi Arabia; muqtadernano@gmail.com; 7 Department of Biomedical Science, College of Pharmacy, Shaqra University, Al-Dawadmi Campus, Dawadmi 11961, Saudi Arabia; drswamy@su.edu.sa

**Keywords:** pyrazolone derivatives, molecular docking, antimicrobial, antiepileptic, ^1^H NMR, mass spectrometry

## Abstract

**Background/Objectives:** Epilepsy is characterized by unpredictable seizures and drug resistance, along with rising antimicrobial resistance (AMR), highlighting the urgent need for innovative dual-action therapies. This study aimed to design, develop, and evaluate novel pyrazolone derivatives for a dual antimicrobial and antiepileptic potential. **Methods:** Novel pyrazolone derivatives were designed, synthesized (using 2,4-dinitrophenylhydrazine/semicarbazide condensation with ethyl acetoacetate), and evaluated through molecular docking against antimicrobial (4URM, 3FYV, 3FRA) and neuronal targets (4COF, 5TP9, 5L1F). The in vitro antimicrobial activity was assessed against Gram-positive (*S. aureus*) and in vitro Gram-negative (*E. coli*, *P. aeruginosa*) strains via agar cup plate assays, while in vivo antiepileptic efficacy was tested in a PTZ-induced seizure model in Swiss albino mice. **Results:** Compound IIa showed potent dual activity, inhibiting *E. coli* (9 mm zone at 80 μg/mL) and *S. aureus* (9.5 mm at 80 μg/mL), alongside a significantly delayed seizure onset in the PTZ-induced mouse model (100% survival rate, 45 sec delayed seizure onset, *p* < 0.001). Compounds Ia and Id showed selective activity against *E. coli* (6 mm at 80 μg/mL) and P. aeruginosa (7 mm at 80 μg/mL), respectively. Docking studies revealed that compound IIa has a superior binding affinity (−7.57 kcal/mol for 3FYV) compared to standards, driven by hydrogen bonds (SER X: 49) and hydrophobic interactions (LEU X: 20). **Conclusions:** This study presents a novel approach by proposing a rationally designed pyrazolone scaffold exhibiting both antimicrobial and antiepileptic activity, which integrates in silico modeling with experimental validation. Compound IIa emerged with preliminary dual biological activities, exhibiting strong antibacterial activity, a superior binding affinity toward both bacterial and neuronal targets, and notable seizure prevention in vivo. These findings show the potential of multifunctional pyrazolone derivatives as a new treatment strategy for addressing drug-resistant infections linked to epilepsy and support further optimization toward clinical development.

## 1. Introduction

Epilepsy causes unexpected, unprovoked seizures due to aberrant brain neuronal activity. Symptoms of seizures include sensory, motor, autonomic, and psychological effects [1,2]. This ailment affects cognitive, physical, and mental health and is typically linked to memory and attention issues, anxiety, depression, migraines, and sleep disorders. Some cases are hereditary, while others are caused by infections and brain trauma [3]. Epilepsy is a set of neurophysiological diseases with many causes and symptoms. Almost all epilepsy has behavioral signs. Epilepsy can induce episodic or non-episodic seizures in all ages [4]. The WHO considers epilepsy a major public health issue, affecting over 50 million people worldwide. Over the past few decades, epileptic medicine development has focused on no seizures and no adverse effects [5]. Despite several breakthroughs in antiepileptic drug (AED) development, adverse effects frequently cause treatment failure. Antiepileptic drugs are chosen based on the seizure type, age, health, and drug interactions [6]. The most common side effects of AEDs are ataxia, blurred vision, diplopia, cognitive impairment, mood/behavior disturbance, headache, dizziness, fatigue, gastrointestinal irritation, visual field loss, hyponatremia, insomnia, vitamin D/folate deficiency, somnolence, weight gain, tremor, hair loss, hirsutism, and more. Bad metabolites produced during the metabolic process induce many side effects. The metabolism changes the medication using specialized enzymes [7]. More effective, side-effect-free antiepileptic drugs are needed. FDA-approved AEDs target calcium and sodium channels, reduce Glutamate receptor-regulated synaptic activation, and enhance GA-BAergic effects by inhibiting GABA reuptake, affecting the GABA-A receptor allosteric site, or inhibiting GABA-AT [8].

Antimicrobial resistance (AMR) has become a major global health concern, making it difficult to treat bacterial infectious diseases. AMR poses a challenge to the efficient prevention and management of a growing number of diseases brought on by bacteria, parasites, viruses, and fungi. Drug-resistant infections are mostly caused by the overuse and abuse of antibiotics in humans, animals, and plants [9]. Self-medication, improper prescriptions, insufficient therapy, using leftover antibiotics without a doctor’s supervision, and partial antibiotic treatment are examples of antibiotic overuse and abuse. Antibiotic enzymatic modification or degradation, the restriction of antibiotic entry into cells to prevent accumulation, changes to metabolic pathways, the modification of binding sites such as ribosomes to decrease drug effectiveness, and increased activity of efflux pumps that remove antibiotics from cells before they can reach adequate levels are typical resistance mechanisms [10,11,12]. Additionally, bacteria can form surface-bound colonies known as biofilms; such a formation significantly enhances resistance by restricting the penetration of antibiotics and promoting microbial survival under adverse conditions [13]. According to the WHO (2024), AMR has contributed to 5 million fatalities worldwide and was directly responsible for 1.27 million deaths, while epilepsy affects 1% of the population, with 30% of cases being resistant to drugs. AMR not only causes mortality and disability, but it also has high financial expenses. According to World Bank projections, AMR may lead to USD 1 trillion in increased healthcare expenses by 2050 and USD 1 trillion to USD 3.4 trillion in annual GDP losses by 2030 [14].

Pyrazolone and its derivatives are one of the most important classes of heterocyclic, five-membered compounds containing two adjacent nitrogen atoms, possessing an additional carbonyl group [15]. These are important moieties for combinatorial and medicinal chemistry due to their easy preparation and have a wide range of pharmacological activities, like antiepileptic [16,17,18], anti-inflammatory [19,20], antibacterial, antifungal [21,22,23], antioxidant [24,25], antitubercular [26,27], antihyperglycemic [28,29], and anticancer activity [30,31]. According to the literature review, pyrazolone molecules have a wide range of pharmacological properties, including antibacterial and antiepileptic properties. The development of single molecules that possess potential dual antimicrobial and antiepileptic properties provides better therapeutic advantages. Targeting both pathways is clinically relevant as epileptic patients often exhibit an increased susceptibility to infections, and dual-action agents may reduce the pill burden and drug–drug interactions. Furthermore, they also provide healthcare professionals with more convenience and possible cost savings. In the present work, we aim to design, perform molecular docking on, and synthesize novel pyrazolone derivatives by treating different hydrazines with ethyl acetoacetate and evaluating their potential dual antimicrobial and antiepileptic activity.

## 2. Results

### 2.1. Chemistry

In this study, novel pyrazolone derivatives were designed, subjected to molecular docking, synthesized, and evaluated for their antibacterial and antiepileptic activity. The results of the FT-IR, ^1^H NMR, and mass spectrometry were used to confirm the structure of the synthesized pyrazolone derivatives. The pyrazolone derivatives were synthesized by reacting 2,4-dinitrophenylhydrazine/semicarbazide with ethyl acetoacetate, followed by condensation with different aromatic aldehydes in the presence of a base.

The infrared spectra of compounds Ia, Ib, Ic, and Id showed a peak of C=N stretching at 1580–1590 cm^−1^, C-N stretching at 1130–1140 cm^−1^, C=O stretching at 1617 cm^−1^, and C=C stretching at 1510–1515 cm^−1^. Compounds IIa, IIb, IIc, and IId showed a peak of N-H stretching at 3330–3410 cm^−1^, C-N amide stretching at 1430–1460 cm^−1^, C=N stretching at 1590–1597 cm^−1^, C-N stretching at 1075–1167 cm^−1^, C=O stretching at 1640–1690 cm^−1^, and C=C stretching at 1510–1520 cm^−1^. The FT-IR spectra of the synthesized compounds I (a–d) and II (a–d) are shown in Supplementary Figures S1A–S8A.

^1^H NMR spectra of compounds Ia, Ib, Ic, Id, IIa, IIb, IIc, and IId, recorded in DMSO-d6 (400 MHz), confirmed the expected proton environment. Compound Ia showed a signal at δ 2.01 totaling six protons (3.00 + 3.02 = 6.02), which may be attributed to trace impurities in the deuterated solvent, DMSO-d_6_, specifically the residual protiated DMSO (DMSO-d_5_). Notably, DMSO-d_5_ commonly appears in the ^1^H NMR spectrum as a residual peak at 2.1–2.4 ppm [32,33]. In the NMR spectrum, the signal of the aromatic proton present between two nitro groups undergoes weak intermolecular interactions and hence is not fully integrated.

There was a singlet at δ 10.12 for the hydroxyl proton (-OH) and a singlet at δ 3.35 for the methyl (-CH_3_) proton. The aromatic protons with vinylic protons (δ value shifts because of the aromatic environment) were observed as a multiplet between δ 6.80 and 8.80. For compounds Ib and Ic, the spectra showed singlets at δ 10.13 and δ 10.20 for the -OH proton, respectively. Compound Id exhibited a singlet at δ 3.38 for the methoxy (-OCH_3_) group [34].

^1^H NMR spectra of compounds IIa, IIb, IIc, and IId (400 MHz, DMSO): Compound IIa also showed a signal at δ 2.13, indicating the presence of a residual solvent peak, with the -CH_3_ proton appearing at δ 3.35, the hydroxyl (-OH) proton appearing at δ 6.90, and the amino group (-NH_2_) appearing at δ 10.10. The aromatic protons with vinylic protons (δ value shifts because of the aromatic environment) appeared in the range of δ 7.60–8.60. IIb lacked the OH signal, while IIc showed the OH proton at δ 6.80. IId exhibited a singlet for the -OCH_3_ group at δ 3.80 and a singlet for the -OH proton at δ 6.90, as shown in Supplementary Figures S1B–S8B. The FT-IR, ^1^H NMR, and mass spectra of the synthesized compounds Ia, Ib, Ic, Id, IIa, IIb, IIc, and IId are shown in Supplementary Figures S1–S8.

### 2.2. Synthesis of 2-(2,4-Dinitrophenyl)-5-methyl-2,4-dihydro-3H-pyrazol-3-one (I)

**Figure 1:**
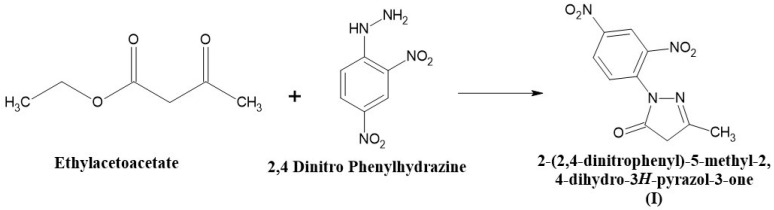
The scheme for the synthesis of 2-(2,4-Dinitrophenyl)-5-methyl-2,4-dihydro-3H-pyrazol-3-one (I).

An equimolar quantity of ethyl acetoacetate (0.1 moles) and 2, 4, dinitro phenylhydrazine (0.1 moles) was mixed and heated in a boiling water bath with occasional stirring for 1 h in a fume cupboard. After the completion of the reaction, a heavy syrup was formed. The heavy syrup was then cooled to room temperature, and 250 mL of diethyl ether was added with vigorous stirring. The product was formed, filtered, and washed with a small amount of diethyl ether. The compound was then dried and recrystallized from 95% ethanol. The melting point was found to be 94 °C. The compound (II) synthesis scheme is shown in Figure 1.

### 2.3. Synthesis of 3-Methyl-5-oxo-4,5-dihydro-1H-pyrazole-1-carboxamide (II)

**Figure 2:**
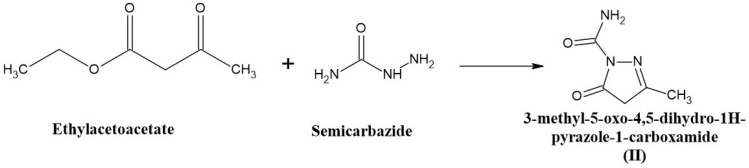
The scheme for the synthesis of 3-methyl-5-oxo-4,5-dihydro-1H-pyrazole-1-carboxamide.

An equimolar quantity of ethyl acetoacetate (0.1 moles) and semicarbazide (0.1 moles) was mixed and heated in a boiling water bath with occasional stirring for 1 h in a fume cupboard. After the completion of the reaction, a heavy syrup was formed. The reaction mixture was then cooled to room temperature, and 250 mL of water was added with vigorous stirring. The product was formed, filtered, and washed with a small amount of diethyl ether. The compound was then dried and recrystallized from 95% ethanol. The melting point was found to be 264–266 °C. The compound (II) synthesis scheme is shown in Figure 2.

### 2.4. Synthesis of 2-(2,4-Dinitrophenyl)-5-methyl-2,4-dihydro-3H-pyrazol-3-one Derivatives (Ia, Ib, Ic, and Id)

**Figure 3:**
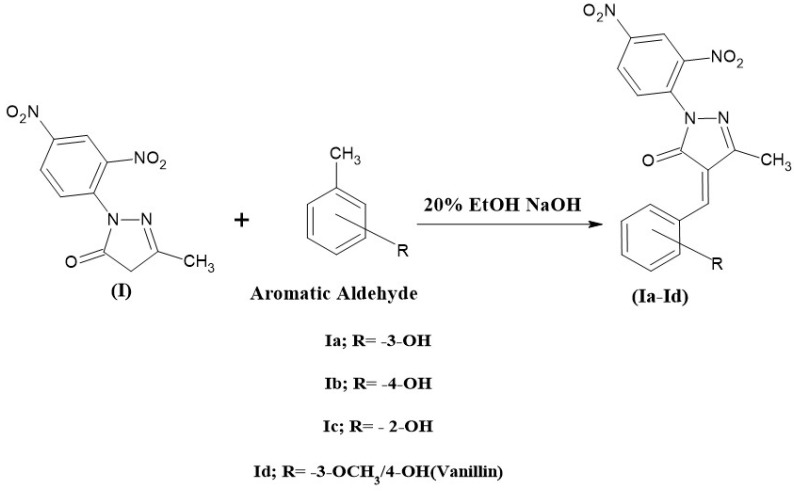
The scheme for the synthesis of 2-(2,4-dinitrophenyl)-5-methyl-2,4-dihydro-3H-pyrazol-3-one derivatives.

Then, 0.01 mole of compound I and 50 mL of the freshly prepared 20% ethanolic sodium hydroxide solution was collected in a 250 mL beaker, and the mixture was stirred for 30 min in a magnetic stirrer. Then, 0.01 mole of aromatic aldehyde was added (vanillin, 2-OH, 3-OH, and 4-OH benzaldehyde) and stirred for 8 h. The reaction mixture was then poured into crushed ice and neutralized with dilute HCl to precipitate the product, which was then kept in a refrigerator overnight. The product was filtered, dried, and recrystallized with a suitable solvent. The compound (Ia, Ib, Ic, and Id) synthesis scheme is shown in Figure 3.

3-Hydroxy-3-(3-hydroxybenzylidene)-1-(2,4-dinitrophenyl)-4-methylpyrazol-5-one (Ia). Prepared from I Pale Yellow, 60% yield, m.p. 150–152 °C. IR (KBr, νmax cm^−1^): 1587 (C=N), 1139 (C-N), C=O (1617), C=C (1512), O-H (3438) N-O (1331); ^1^H-NMR (400 MHz, DMSO-d6): δ 2.01 (3H, CH_3_), 6.8–8.8 (7H, Ar-H), 3.35(1H, C=CH), 10.12 (1H, O-H); MS: *m*/*z* = 367, 301, 237.

Hydroxy-3-(3-hydroxybenzylidene)-1-(2,4-dinitrophenyl)-4-methylpyrazol-5-one (Ib). Prepared from I Colorless Solid, 65% yield, m.p. 160–162 °C. IR (KBr, νmax cm^−1^): 1590 (C=N), 1133 (C-N), C=O (1617), C=C (1513), O-H (3438), N-O (1332); ^1^H-NMR (400 MHz, DMSO-d6): δ 2.13 (3H, CH_3_), 7.64–8.87 (7H, Ar-H), 3.35 (1H, C=CH), 10.13 (1H, O-H); MS: *m*/*z* = 305, 261, 172, 137.

2-Hydroxy-3-(3-hydroxybenzylidene)-1-(2,4-dinitrophenyl)-4-methylpyrazol-5-one (Ic). Prepared from I Yellowish White, 55% yield, m.p. 164- 166 °C. IR (KBr, νmax cm^−1^): 1588 (C=N), 1138 (C-N), C=O (1617), C=C (1514), O-H (3433), N-O (1333); ^1^H-NMR (400 MHz, DMSO-d6): δ 2.13 (3H, CH_3_), 7.28–8.89 (7H, Ar-H), 3.35 (1H, C=CH), 10.20 (1H, O-H); MS: *m*/*z* = 367, 301.

3-Methoxy-4-Hydroxy-3-(3-hydroxybenzylidene)-1-(2,4-dinitrophenyl)-4-methylpyrazol-5-one (Id). Prepared from I Yellowish White, 50% yield, m.p. 144–146 °C. IR (KBr, νmax cm^−1^): 1590 (C=N), 1132 (C-N), C=O (1617), C=C (1515), O-H (3400) C-O-C (1214), N-O (1332); ^1^H-NMR (400 MHz, DMSO-d6): δ 2.14 (3H, CH_3_), 3.88 (3H, OCH_3_), 7.40–8.87 (7H, Ar-H), 3.35 (1H, C=CH), 10.13 (1H, O-H); MS: *m*/*z* = 301, 261, 172, 137.

### 2.5. Synthesis of 3-Methyl-5-oxo-4,5-dihydro-1H-pyrazole-1-carboxamide Derivatives (IIa, IIb, IIc, and IId)

**Figure 4:**
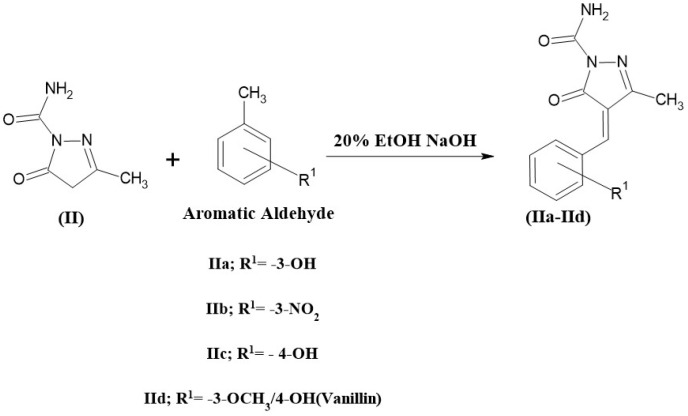
The scheme for the synthesis of 3-methyl-5-oxo-4, 5-dihydro-1H-pyrazole-1-carboxamide derivatives.

A total of 0.01 mole of compound II and 50 mL of the freshly prepared 20% ethanolic sodium hydroxide solution was combined in a 250 mL beaker, and the mixture was stirred for 30 min in a magnetic stirrer. The reaction mixture was mixed with 0.01 mole of aromatic aldehydes (vanillin, 2-OH, 3-OH, and 4-OH benzaldehyde) and stirred more for 8 h. The reaction mixture was then poured into crushed ice and neutralized with dilute HCl to precipitate the product, which was then frozen for the night. The product was filtered, dried, and recrystallized with a suitable solvent [35]. The compound (IIa, IIb, IIc, and IId) synthesis scheme is shown in Figure 4.

3-Methyl-4-[(3-hydroxyphenyl)methylidene]-5-oxo-1H-pyrazole-1-carboxamide (IIa). Prepared from II, Colorless Solid, 60% yield, m.p. 130–132 °C. IR (KBr, νmax cm^−1^): 1451 (C-N of amide), 3339 (N-H), 1597 (C=N), 1163 (C-N), C=O (1675), C=C (1510), O-H (3026); ^1^H-NMR (400 MHz, DMSO-d6): δ 2.13 (3H, CH_3_), 7.69–7.79 (4H, Ar-H), 3.35 (1H, C=CH), 10.1 (2H, NH_2_), 6.9 (1H, O-H); MS: *m*/*z* = 242.

3-methyl-4-[(3-nitrophenyl)methylidene]-5-oxo-4,5-dihydro-1H-pyrazole-1carboxamide (IIb). Prepared from II, Brown Color, 62% yield, m.p. 138–142 °C. IR (KBr, νmax cm^−1^): 1438 (C-N of amide), 3410 (N-H), 1597 (C=N), 1075 (C-N), 1691(C=O), C=C (1530), N-O (1350); ^1^H-NMR (400 MHz, DMSO-d6): δ 2.13 (3H, CH_3_), 7.69–7.79 (4H, Ar-H), 6.90 (1H, C=CH), 8.75 (s, 2H, NH_2_)); MS: *m*/*z* = 273, 167.

3-Methyl-4-[(4-hydroxyphenyl)methylidene]-5-oxo-1H-pyrazole-1-carboxamide (IIc). Prepared from II, Colorless Solid, 68% yield, m.p. 152–154 °C. IR (KBr, νmax cm−1): 1447 (C-N of amide), 3408–(N-H), 1595 (C=N), 1167 (C-N), C=O (1657), C=C (1514), O-H (3305); ^1^H-NMR (400 MHz, DMSO-d6): δ 2.14 (3H, CH_3_), 7.70–7.79 (4H, Ar-H), 6.90 (1H, C=CH), 8.60 (2H, NH_2_), 10.20 (1H, O-H); MS: *m*/*z* = 242.

3-Methyl-4-[(3-methoxy-4-hydroxyphenyl)methylidene]-5-oxo-1H-pyrazole-1-carboxamide (IId). Prepared from II, Colorless Solid, 64% yield, m.p. 148–150 °C. IR (KBr, νmax cm^−1^): 1449 (C-N of amide), 3408 (N-H), 1597 (C=N), 1143 (C-N), 1648 (C=O), 1517 (C=C), 3366 (O-H), C-O-C (1204); ^1^H-NMR (400 MHz, DMSO-d6): δ 2.02 (3H, CH_3_), δ 3.859 (3H, OCH_3_), 7.3–7.61 (3H, Ar-H), 3.35 (1H, C=CH), 8.78 (2H, NH_2_), 9.78 (1H, O-H); MS: *m*/*z* = 274, 229, 137.

### 2.6. Molecular Docking

In a computer-based drug design, a docking simulation is a crucial analysis. Molecular docking helps elucidate molecular interactions and confirm binding modes. Molecular docking aims to create an ideal balance for how the protein and synthetic molecules relate to each other since it lowers the system’s free energy.

AutoDock 4.2.6 was chosen for its reliable scoring function, capacity to handle flexible ligand docking, and widespread validation for screening heterocyclic compounds against bacterial and neural receptors. The Lamarckian Genetic Algorithm used is especially good for exploring conformational space and predicting binding modes important for dual-target therapies. 

**Figure 5:**
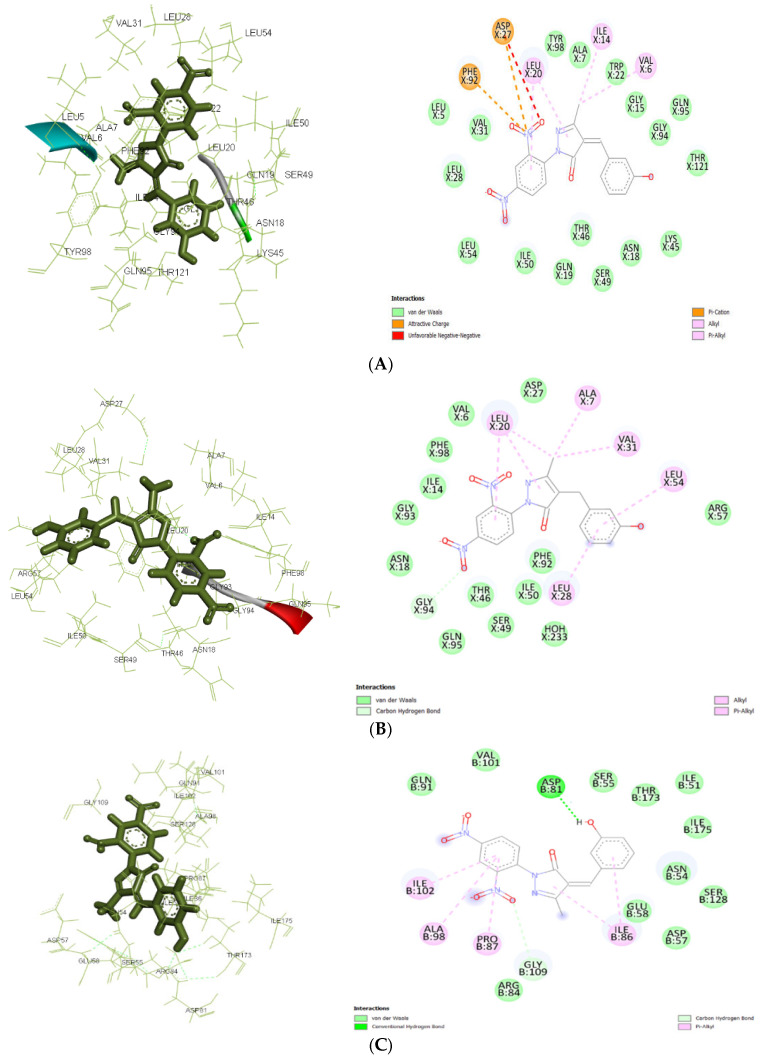
(**A**): Docking interactions of ligand Ia with antibacterial targets. (**A**): The 2D and 3D interactions with *S. aureus* DHFR (3FRA). (**B**): Docking interactions of ligand Ia with antibacterial targets. The 2D and 3D interactions with *E. coli* DHFR (3FYV). (**C**): Docking interactions of ligand Ia with antibacterial targets. The 2D and 3D interactions with *DNA gyrase* (4URM).

**Figure 6:**
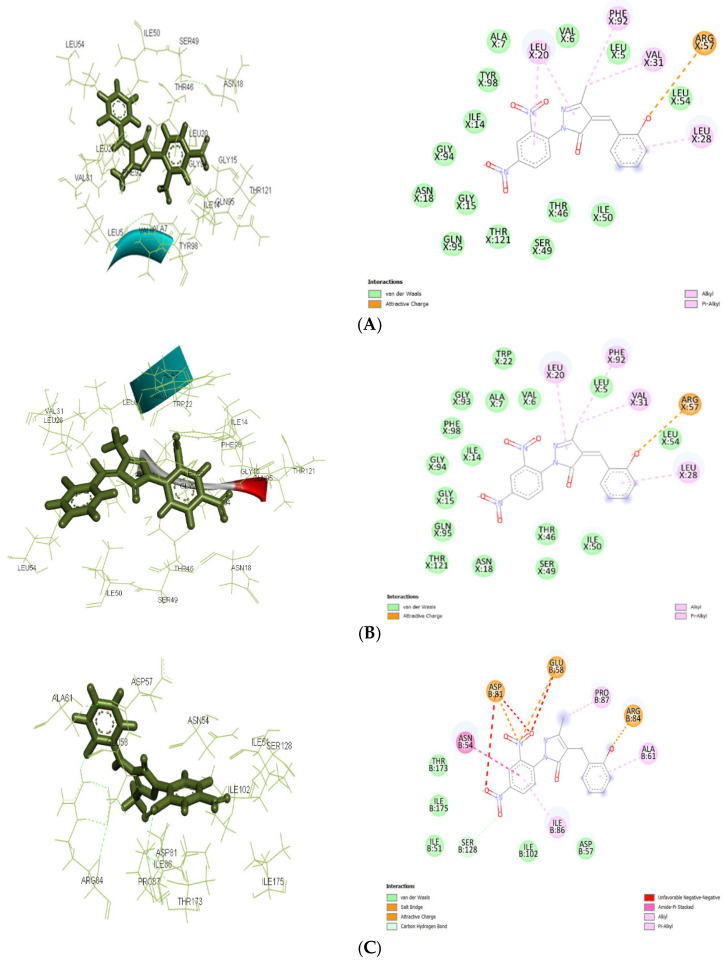
The 2D and 3D docking interactions for ligand Ic with antibacterial targets (**A**) against 3FRA. The 2D and 3D docking interactions for ligand Ic with antibacterial targets (**B**) against 3FYV. (**C**): The 2D and 3D docking interactions for ligand Ic with antibacterial targets (**C**) against 4URM.

**Figure 7:**
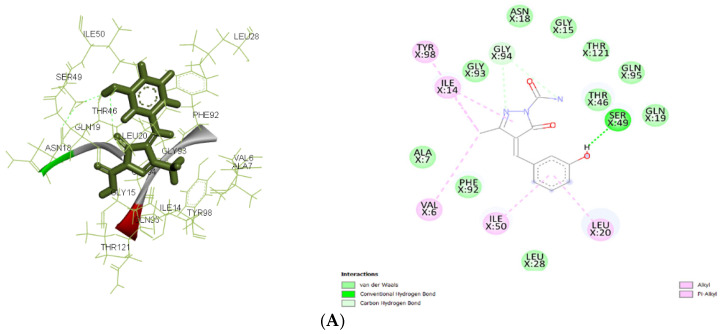

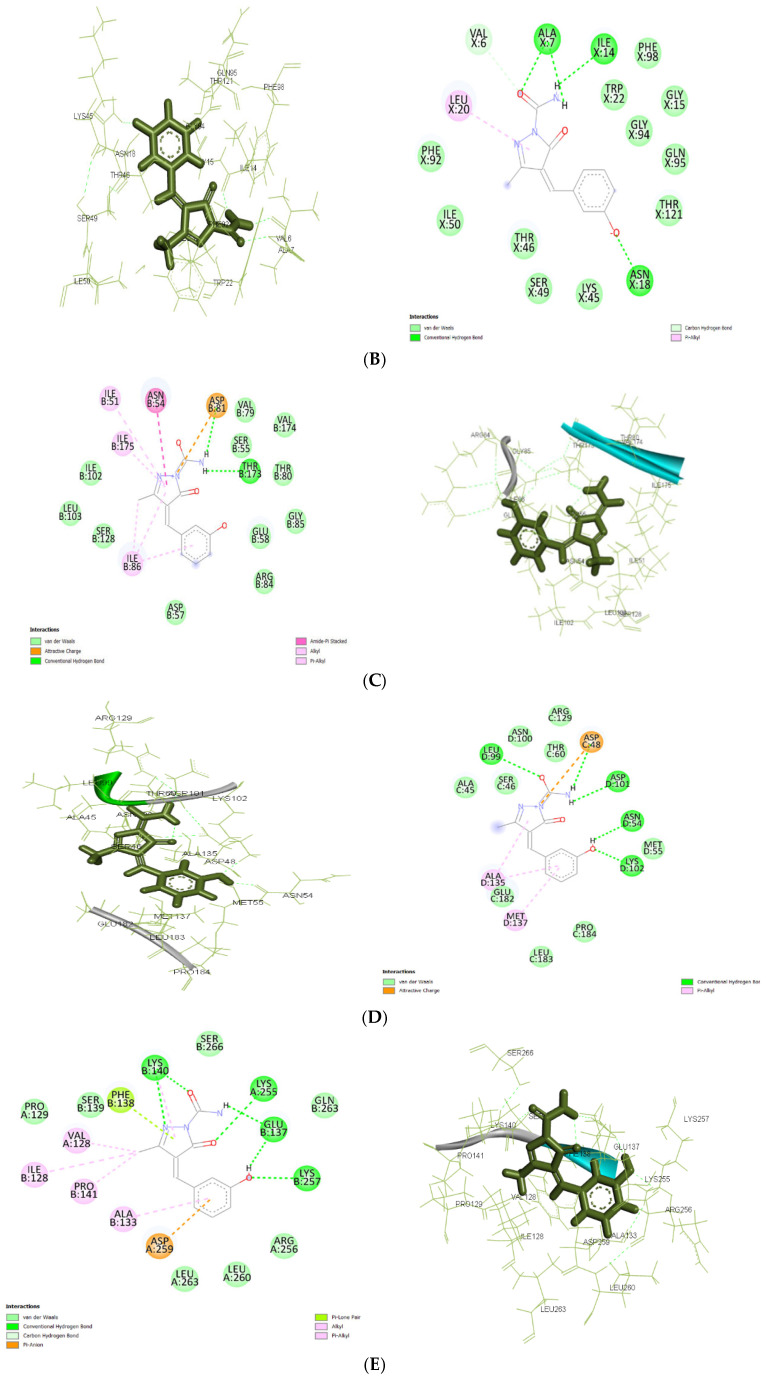

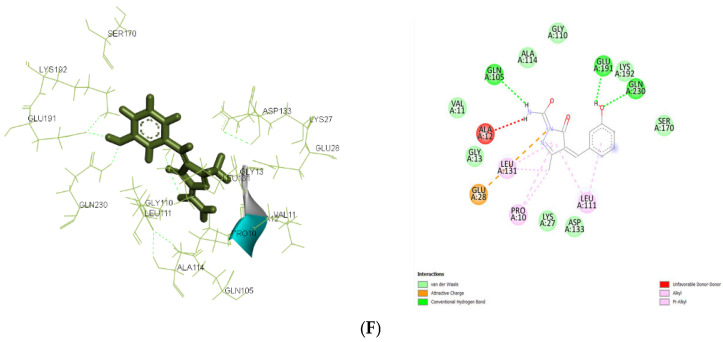
(**A**): The 2D and 3D docking interactions for ligand IIa with antibacterial and antiepileptic targets against 3FRA. (**B**): The 2D and 3D docking interactions for ligand IIa with antibacterial and antiepileptic targets against 3FYV. (**C**): The 2D and 3D docking interactions for ligand IIa with antibacterial and antiepileptic targets against 4URM. (**D**): The 2D and 3D docking interactions for ligand IIa with antibacterial and antiepileptic targets against 4COF. (**E**): The 2D and 3D docking interactions for ligand IIa with antibacterial and antiepileptic targets against 5TP9. (**F**): The 2D and 3D docking interactions for ligand IIa with antibacterial and antiepileptic targets against 5L1F.

In the current study, AutoDock 4.2.6 was used to analyze the interaction between pyrazolone derivatives and several protein targets: 4URM (DNA gyrase), 3FYV and 3FRA (dihydrofolate reductase), 4COF (GABA receptor subunit beta-3), 5TP9 (NMDA receptor subunits NR1 and NR2A), and 5L1F (Glutamate receptor 2). DNA gyrase (4URM) adds negative supercoils to DNA, necessary for transcription and replication. The 2D and 3D docking interactions for ligand Ia against 3FRA, 3FYV, and 4URM are shown in Figure 5A, Figure 5B, and Figure 5C, respectively. The 2D and 3D docking interactions for ligand Ic against 3FRA, 3FYV, and 4URM are shown in Figure 6A, Figure 6B, and Figure 6C, respectively. The 2D and 3D docking interactions for ligand IIa against 3FRA, 3FYV and 4URM, 4COF, 5TP9, and 5L1F are shown in Figure 7A, Figure 7B, Figure 7C, Figure 7D, Figure 7E, and Figure 7F, respectively. Dihydrofolate reductase (3FYV, 3FRA) is essential for DNA synthesis and repair through the tetrahydrofolate pathway. DNA gyrase (4URM) is essential for maintaining the physiological stability and active state of DNA via supercoiling and helps in forming DNA double strands. The GABA receptor subunit beta-3 (4COF) is involved in inhibitory neurotransmission, playing a role in anxiety and epilepsy. The Nmda receptor subunits NR1 and NR2A (5TP9) are key for synaptic plasticity and memory, implicated in epilepsy and Alzheimer’s disease. Glutamate receptor 2 (5L1F), part of the AMPA receptor subtype, is crucial for fast synaptic transmission and cognitive functions. Many scientists have focused on the mentioned proteins when creating innovative antibacterial and antiepileptic drugs [36,37,38].

Molecular docking results showed significant mechanistic differences in the binding interactions and affinity between designed molecules and control ligands or standard inhibitors of both antibacterial and antiepileptic targets. For the antimicrobial target 3FRA, compound IIa showed the highest binding affinity (−7.499 kcal/mol), followed by Ia (−7.037 kcal/mol), Id (−6.889 kcal/mol), and the control ligand (−6.67 kcal/mol). The strongest binding affinity of IIa was significantly due to the strong Van der Waals interactions with GLN X: 19, GLN X: 95, THR X: 46, and THR X: 121, along with conventional hydrogen bonding with SER X: 49. Hydrophobic stabilization through alkyl/pi-alkyl interactions with LEU X: 20, ILE X: 50, and VAL X: 6 further enhanced its stability. Van der Waals interactions with key amino acid residues including THR X: 121, GLY X: 94, and ASN X: 18, as well as Pi–cation interactions with ASP X: 27 and PHE X: 92, were responsible for ligand Ia’s highest binding affinity. Id’s moderate binding stability was further influenced by the hydrogen bonding with ALA X: 7 and Pi–Pi stacking interactions with PHE X: 92. Because of fewer electrostatic interactions, the control ligand demonstrated less stabilization even though it formed hydrogen bonds with ASN X: 18 and GLN X: 19.

For 3FYV, ligand IIa exhibited a higher binding affinity (−7.57 kcal/mol) than Ia (−7.025 kcal/mol), Id (−6.807 kcal/mol), and the control (−6.73 kcal/mol). The strongest binding interaction is due to the Van der Waals interactions with amino acids, such as THR X: 121, GLN X: 95, and GLY X: 94, in addition to interactions with ASN X: 18 and ALA X: 7 through conventional hydrogen bonding. Hydrophobic packing through alkyl/pi–alkyl interactions with LEU X: 20 further reinforced its stability.

In addition to carbon–hydrogen bonding with GLY X: 94, ligand Ia showed an identical binding affinity and strong Van der Waals interactions with ARG X: 57, ASP X: 27, and PHE X: 98. Id used a similar strategy, depending on the hydrophobic interactions with PHE X: 92 and LEU X: 54 and hydrogen bonding with LEU X:28. Despite establishing hydrogen bonds with ASN X: 18 and GLN X: 19, the control ligand’s stability was comparatively lower because of less Van der Waals and electrostatic interactions.

In 4URM, the control ligand showed the highest binding energy (−7.82 kcal/mol), followed by IIa (−6.838 kcal/mol), Ia (−6.534 kcal/mol), and Id (−6.273 kcal/mol). Along with conventional hydrogen bonds with GLY B: 110 and LYS B: 93, the binding of the control molecule was due to Van der Waals interactions with GLY B: 85, THR B: 173, and ASP B: 81. Ligand IIa demonstrated a somewhat reduced binding affinity even though it stabilized alkyl/pi–alkyl interactions with ILE B: 86 and ILE B: 175 and formed a salt bridge with ASP B: 81. The vast hydrogen bond network shown in the control ligand was absent from Ia and Id, which instead depended on Van der Waals and electrostatic interactions.

In 5L1F, ligand IIa (−7.409 kcal/mol) showed a stronger binding affinity than the control (−7.190 kcal/mol) because of extensive hydrogen bonding with GLN A: 105, GLU A: 191, and GLN A: 230. The further electrostatic stabilization between the ligand and proteins is significantly due to the Pi–cation interaction with GLU A: 28 and hydrophobic interactions with LEU A: 111, PRO A: 10, and LEU A: 131, while the control ligand relied more on Pi-Pi stacking interactions, making its binding less stable.

For 4COF, ligand IIa showed a higher binding affinity (−7.474 kcal/mol) than the control (−5.33 kcal/mol). Multiple stabilizing hydrogen bonds with LEU D: 99, ASP D: 101, ASN D: 54, and LYS D: 102, as well as an electrostatic salt bridge interaction with ASP C: 48, were responsible for this difference. The lower affinity resulted from weaker electrostatic interactions and fewer hydrogen bonds in the control molecule.

Similarly, in 5TP9, ligand IIa displayed a better binding affinity (−6.55 kcal/mol) than the control (−5.84 kcal/mol). Vander Waal interactions, π stacking with PHE B: 138, and strong hydrogen bonds with LYS B: 140, GLU B: 137, and LYS B: 257 increase the binding affinity compared to the control. Despite generating Pi–cation stacking with His B: 273 and TYR B: 144, the control ligand’s overall stabilization was poorer.

The docking results of the designed ligands were compared with the co-crystallized ligand or control ligands of the respective target, such as I2H (3FRA), XCF (3FYV), XAM (4URM), 7H0 (5TP9), 6ZP (5L1F), and 4COF (BEN). The control ligands were selected based on their known binding affinities and documented interactions with the target proteins. The docking results of control ligands with target proteins help to confirm the specificity of the binding interactions, distinguishing true binding events from nonspecific ones. It is also essential for validating the docking protocol. By comparing the docking results of designed compounds with these control ligands, we can evaluate the efficacy and selectivity of the new compounds, thereby enhancing the credibility of our findings. The docking scores of designed ligands are given in Table 1.

### 2.7. In Silico Drug-Likeness Prediction

Drug-likeness reflects a compound’s potential to become an orally active drug, based on its physicochemical and structural features. To assess this, Lipinski’s Rule of Five (Ro5) was applied using the SwissADME web server. The Ro5 states that a compound is more likely to exhibit favorable oral bioavailability if it meets the following criteria: a molecular weight ≤ 500 Dalton, a Log P ≤ 5, no more than 5 hydrogen bond donors, no more than 10 hydrogen bond acceptors, and a molar refractivity between 40 and 130 [39,40]. All the synthesized compounds (Ia–Id and IIa–IId) met these criteria, with no or minimal violations, as summarized in Table 2. It is important to note that while the Ro5 provides a useful filter, several orally bioavailable drugs—especially natural products, antibiotics, and peptide-based molecules—may violate one or more of these rules. Such exceptions are often absorbed via active transport mechanisms, such as peptide transporters (PEPT1/2), organic anion transporters (OATs), or carrier-mediated endocytosis, which circumvent passive diffusion limitations [41,42].

In addition to Lipinski’s rules, other drug-likeness indicators, such as the topological polar surface area (TPSA) and molar refractivity, were considered. A TPSA under 140 Å^2^ generally favors intestinal absorption, and all compounds except Id were within this threshold. Despite a minor violation in compound Id, the overall drug-likeness profile suggests favorable pharmacokinetic behavior [43]. In silico predictions indicated low toxicity; comprehensive in vivo toxicity studies will be performed in subsequent optimization phases.

### 2.8. Antibacterial Activity:

All synthesized novel pyrazolone moieties were examined for their antibacterial activity. The zone of inhibition (mm) for the antibacterial activity data is expressed and summarized in Table 3. Compound IIa showed a zone of inhibition of 7 mm and 9 mm for *E. coli* at 40 μg/mL and 80 μg/mL. It also showed zones of inhibition of 6 mm, 8 mm, and 9.5 mm for *S. aureus* at 40, 60, and 80 μg/mL. Compound Ia showed a zone of inhibition of 6 mm for *E. coli* at 80 μg/mL. Compound Id showed a zone of inhibition of 6 mm and 7 mm for *P. aeruginosa* at 60 and 80 μg/mL. Only compounds exhibiting measurable zones of inhibition are reported; remaining derivatives (Ib, Ic, IIb, IIc, and IId) showed no activity at concentrations up to 80 µg/mL. The SAR study revealed that the presence of hydroxyl (-OH) groups is essential for desired antibacterial activity. Their importance is probably because of their capacity to form hydrogen bonds, which might enhance interactions with target proteins. The modest activity shown by compounds with methoxy (-OCH_3_) and hydroxyl (-OH) groups indicates the significance of the presence of electron-donating substituents to enhance bioactivity. The enhanced dual activity of IIa is likely due to its balanced hydrophobicity, hydrogen bonding potential, and optimal steric fit within the active sites of both target classes. The presence of a nitro group (-NO_2_) and a withdrawing group showed decreased activity. To validate these tendencies, further thorough research is necessary. Although the agar diffusion method provided initial activity profiles, future work will include MIC/MBC determinations to quantitatively assess antibacterial efficacy.

Compared to previously reported pyrazolone-based antibacterial drugs [21], com-pound IIa showed broader spectrum activity, particularly against *S. aureus*, most likely due to improved hydrophobicity and hydrogen-bonding abilities. Similarly, its antiepileptic efficacy is consistent with previous findings on pyrazole-derived TRPC3 inhibitors [17], albeit with a unique dual-action profile.

### 2.9. Antiepileptic Activity

**Figure 8:**
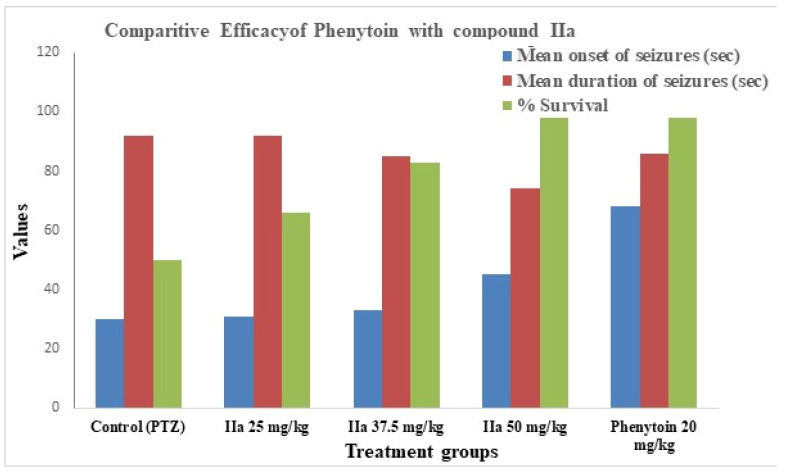
Comparative efficacy of Phenytoin and compound IIa.

We selected compound IIa for in vivo antiepileptic testing based on its highest docking affinity against key antiepileptic targets and notable antibacterial activity, suggesting its potential as a dual-action candidate [docking score [−7.474 (4COF), −6.55 (5TP9), and −7.409 (5L1F)] and examined its antiepileptic activity using the PTZ-induced convulsion method at three different concentrations. At 50 mg/kg, it shows moderate activity; i.e., it increases the mean onset of seizures and decreases the mean duration of seizures. The survival rate is 100% at a dose of 50 mg/kg. The results of the antiepileptic activity are shown in Table 4, and the comparative efficacy of compound II a vs. Phenytoin in PTZ-induced seizures is shown in Figure 8.

In future studies, although docking predicts strong binding, future enzyme inhibition assays will be conducted to experimentally validate target engagement.

## 3. Discussion

The current study aims to investigate pyrazolone derivatives as multifunctional small compounds with preliminary dual biological activity, namely antibacterial and antiepileptic properties, using an integrated in silico–in vitro–in vivo evaluation technique. This strategy tackles two clinically significant challenges: antimicrobial resistance and drug-resistant epilepsy, both of which are characterized by polypharmacy and drug–drug interactions.

### 3.1. Synthesis and Structural Confirmation

The pyrazolone compounds were produced by condensation processes and validated by FT-IR, ^1^H NMR, and mass spectrometry. The spectral results corresponded with the expected functional groups, demonstrating the presence of hydrazine and aldehyde components without degradation.

### 3.2. Justification of Dual-Action Potential

The evidence from many experimental tiers converges to support the dual-action claim. Compound IIa has substantial binding affinities against antibacterial (3FYV: −7.57 kcal/mol; 3FRA: −7.50 kcal/mol) and antiepileptic targets (4COF: −7.47 kcal/mol; 5L1F: −7.41 kcal/mol), with interactions comparable or superior to conventional drugs. IIa demonstrated considerable antibacterial activity in vitro against clinically relevant pathogens and significant seizure prevention in the PTZ paradigm. The pyrazolone core enables multi-target engagement by providing hydrogen bond acceptor/donor motifs and π-system geometries that are compatible with enzyme and receptor binding sites. While translational validation is required, this preliminary dual efficacy marks an important step toward multifunctional medicines that reduce polypharmacy in comorbid populations.

### 3.3. Structure–Activity Relations and Antibacterial Activity

Compound IIa proved to be the most effective antibacterial candidate among the produced compounds, particularly against *Escherichia coli* and *Staphylococcus aureus*. The observed activity appears to be controlled by a balanced combination of hydrogen bonding capability, moderate lipophilicity, and an appropriate steric fit, all of which improve interactions with bacterial enzymes. A structure–activity relationship (SAR) study indicates that hydroxyl-substituted aromatic rings enhance antibacterial effectiveness, most likely by enhancing hydrogen bonding with critical residues in bacterial targets such as dihydrofolate reductase and DNA gyrase. In contrast, derivatives with strong electron-drawing nitro groups showed lower antibacterial activity, presumably due to decreased membrane permeability or adverse electrical effects.

Although the agar cup plate approach offered an initial qualitative assessment of antibacterial activity, the results should be taken as preliminary, and quantitative MIC/MBC measurements are needed in future studies. Nonetheless, when compared to previously reported pyrazolone-based antimicrobial drugs, compound IIa exhibited a broader antibacterial response profile, highlighting the need for more modification.

### 3.4. Antiepileptic Activity and In Vivo Relevance

The antiepileptic efficacy of compound IIa in the PTZ-induced seizure model was dose-dependent, with a delayed seizure onset, reduced seizure duration, and 100% survival at the highest tested dose (50 mg/kg). These data suggest that IIa may alter neuronal excitability but at a lower efficacy than the conventional medication Phenytoin. Importantly, the PTZ model largely reflects the seizure threshold regulation via GABAergic and glutamatergic pathways; thus, any anticonvulsant efficacy observed should be regarded as preliminary. Additional seizure models, such as maximum electroshock (MES) and chronic kindling models, would be needed to fully evaluate the antiepileptic potential and translational significance.

### 3.5. Molecular Docking and Mechanistic Insights

Molecular docking studies revealed mechanistic explanations for the reported biological actions. Compound IIa had high binding affinities for major antibacterial targets (3FYV and 3FRA) and neural receptors linked to epilepsy (4COF, 5TP9, and 5L1F). Importantly, docking data were compared to common reference drugs—Levofloxacin for antibacterial targets and Phenytoin for antiepileptic targets—to ensure the validity of the docking procedure. IIa’s improved or equivalent binding interactions with control ligands were predominantly mediated by hydrogen bonding, van der Waals forces, and hydrophobic contacts, indicating potential multi-target engagement.

However, docking simulations are essentially based on static protein conformations and simplistic solvation models. Thus, while docking data provide useful mechanistic theories, an experimental target validation, such as enzyme inhibition experiments and receptor binding investigations, is required to confirm these interactions.

### 3.6. Drug-Likeness, Safety Concerns, and Study Limitations

In silico ADME predictions revealed that all synthesized compounds, including IIa, largely adhere to Lipinski’s Rule of Five, indicating a positive oral drug-likeness. While these projections indicate a minimal toxicity risk, comprehensive in vivo toxicity and safety tests were outside the scope of the current investigation, posing a significant constraint. Acute, sub-chronic, and chronic toxicity tests, as well as pharmacokinetic profiling, will be required before proceeding with further preclinical research.

Another limitation of this study is the limited scope of the biological examination, specifically the use of a single seizure model and qualitative antimicrobial testing. As a result, the dual-action claim is purposely presented as preliminary, and more mechanistic and translational evidence will be needed to support the multifunctional therapeutic potential.

### 3.7. Novelty and Future Prospects

The new aspect of this study is not the pyrazolone scaffold itself but rather the rational design and systematic evaluation of pyrazolone derivatives for potential dual antibacterial and antiepileptic properties inside a single molecular framework. Such a multimodal approach may eventually help reduce pill loads and drug–drug interactions in epilepsy patients who are susceptible to infections.

Future studies will focus on enzyme inhibition assays to validate docking predictions, MIC/MBC determination for antimicrobial activity, in vivo toxicity and pharmacokinetic profiling, and structural optimization (structural characterization using HRMS and ^13^C NMR) to enhance potency and selectivity. Pyrazolone derivatives may mitigate resistance through novel binding modes and multi-target actions, though future studies will assess susceptibility to common resistance mechanisms such as efflux pumps and β-lactamase inactivation. 

Overall, the present findings demonstrate that pyrazolone derivatives, notably compound IIa, are promising lead multifunctional candidates.

## 4. Materials and Methods

### 4.1. Materials

All chemicals and reagents were purchased from LOBA CHEME PVT Ltd. (Mumbai, India) and S D Fine-CHEM Ltd. (Mumbai, India). We purchased pentylenetetrazole from Yarrow Chem Products in Mumbai, India. To determine the synthesized compound’s melting point, the open capillary technique was employed. ^1^H NMR spectra were recorded using solvent dimethyl sulfoxide (DMSO) on an ECZ 400 high-resolution, multinuclear FT-NMR spectrometer (JOEL, Tokyo, Japan). Waters’ Synapt G2 high-detection mass spectrometry (Waters Corporation, Milford, MA, USA) was used to record the mass spectra.

### 4.2. Methods

#### 4.2.1. Molecular Docking Studies

To understand the binding interactions between the designed pyrazolone ligands and the targeted proteins, molecular docking studies were performed using AutoDock 4.2.6 [44] and compared with the co-crystallized structures of the targeted proteins. The docking studies were carried out according to the following steps.

#### 4.2.2. Target Selection

The present study was focused on antibacterial activity and antiepileptic activity. The Protein Data Bank (PDB) is the largest repository of proteins and their complexes. The three-dimensional structure of target proteins, such as 4URM (DNA gyrase) [45], 3FYV (dihydrofolate reductase) [46], and 3FRA (dihydrofolate reductase) [47], was assessed for antibacterial activity. 4COF (Gamma-Aminobutyric Acid Receptor Subunit Beta-3) [48], 5TP9 (Glutamate receptor ionotropic, NMDA 1 and Glutamate receptor ionotropic, NMDA 2A) [49], and 5L1F (Glutamate receptor 2) [50] for antiepileptic activity were downloaded from the Protein Data Bank (PDB).

#### 4.2.3. Preparation of Ligands

- The desired ligands were drawn using ChemSketch software (ACD/Chem Sketch Freeware).
- The ligands were then converted into three-dimensional PDB format using BIOVIA Discovery Studio software (2021 Client 21.1).
- The PDB files were imported into AutoDock Tools by selecting the ligand file using the “Read Molecule” function.
- Once loaded, the ligand was selected as the molecule for the docking setup by using the appropriate input options.
- Polar hydrogens were added to the ligand structure to enable proper hydrogen bonding during docking.
- Nonpolar hydrogens were merged to simplify the molecular structure.
- Gasteiger charges were calculated and assigned to all atoms of the ligand.
- A torsion tree was set up by detecting the root atom, and the number of rotatable bonds was defined.
- The ligand preparation was finalized by saving the molecule in the required PDBQT format for use in AutoDock docking studies.

#### 4.2.4. Protein Preparation

- The protein preparation was carried out using AutoDock Tools software.
- The proteins were cleaned by removing water and heteroatoms.
- Polar hydrogens were added to the protein structure using the “Add Hydrogens” option available in the edit menu; these appear as white-colored dots on the structure.
- Kollman charges were then assigned to all atoms by selecting the “Add Charges” option under the edit menu.
- The prepared protein was saved in PDBQT format, which is required for further docking studies.

#### 4.2.5. Ligand–Protein Docking Analysis

The grid box for docking simulations was configured using the graphical user interface application AutoDock 4.2.6. We experimented with several different docking pockets and positions before generating the grid based on the best outcomes. The best-docked conformation between the ligand and protein was found using the docking algorithm included with AutoDock Vina. In order to analyze the interactions between the target protein and ligands, the conformations with the most advantageous (lowest) free binding energy were chosen using the Discovery studio visualizer. The selected and designed ligands were docked into the protein’s active site using AutoDock and a conventional procedure. AutoDock Tools, a free graphical user interface for the AutoDock software, was used to conduct the molecular docking research. The grid box for docking was defined with its center coordinates set at X: −28, Y: 9, and Z: 6 Å. The number of grid points was specified as 25 along each axis (X, Y, and Z), with a spacing of 0.3750 Å between points. Using AutoDock Vina routines, one hundred distinct conformations were produced for every ligand, which were then ranked according to their binding energies. We compared docking results of designed compounds with control ligands I2H (3FRA), XCF (3FYV), XAM (4URM), 7H0 (5TP9), 6ZP (5L1F), and 4COF (BEN) [44].

#### 4.2.6. Limitations of Docking Approach

The docking studies presume rigid protein structures and bypass dynamic conformational changes. The solvation effects and entropy contributions were simplified. While docking provides useful binding ideas, the results must be experimentally validated using enzyme tests or structural studies.

### 4.3. In Silico ADME Prediction

Using the SwissADME web server, computational analyses of the synthesized compounds (Ia, Ib, Ic, Id, IIa, IIb, IIc, and IId) were carried out to predict molecular characteristics. Drug-like and non-drug-like molecules can be differentiated by using Lipinski’s Rule of Five. For molecules that satisfy two or more of the following conditions, it predicts a high likelihood of success or failure due to drug-likeness. The molecules should have a molecular mass of less than 500 Dalton, high lipophilicity (shown as Log P less than 5), less than five hydrogen bond donors, less than ten hydrogen bond acceptors, and a molar refractivity of 40–130 [51].

### 4.4. Antibacterial Activity

Using the agar cup plate technique, the antibacterial activity of all newly synthesized compounds was determined against both Gram-positive (Staphylococcus aureus) and Gram-negative (Escherichia coli, Shigella flexnaeri and Pseudomonas aeruginosa) bacteria. *E. coli*, *P. aeruginosa*, and *S. aureus* were selected as model organisms due to their clinical prevalence and representation of key Gram-negative and Gram-positive bacteria [52,53]. Synthesized novel pyrazolone derivatives were tested at different concentrations (40, 60, and 80 μg/mL) by using 20% DMSO. The above bacterial strains were procured from the Adichunchanagiri Institute of Medical Sciences (AIMS). All derivatives at various concentrations were added to the cups produced by a sterile borer and incubated at 37 °C for 24 h. Levofloxacin was used as a standard drug for comparison under the same condition, and 20% dimethyl sulfoxide (DMSO) was used as a control [54].

### 4.5. Antiepileptic Activity

We examined the antiepileptic activity using the pentylenetetrazole (Metrazol)-induced convulsion technique. Healthy Swiss albino mice, weighing approximately 20–28 g, were utilized for this study. They were kept in polypropylene cages and maintained under standard conditions, including a 12 h light and 12 h dark cycle, a temperature of 27 ± 1 °C, and 60% humidity [55,56]. This study was conducted with the approval of the Institutional Animal Ethical Committee, Sri Adichunchanagiri College of Pharmacy, B G Nagara, Karnataka, India. Animal experiments followed ARRIVE guidelines, with protocols approved by SACCP-IAEC (Approval No: SACCP-IAEC/2021-02/47).

In this experiment, 30 Swiss albino mice of either sex were grouped into five groups (*n* = 6). Compound IIa was suspended in Tween 90 and administered at 25 mg/kg, 37.5 mg/kg, and 50 mg/kg for groups II, III, and IV, respectively. After 30 min, PTZ was administered intraperitoneally at a 60 mg/kg dose for group I, and the group was examined for 15 min. Phenytoin was used as a standard drug. According to the Racine scale, motor responses were scored from 0 to 5, with 0 representing immobilization; 2 representing head nodding and partial myoclonus; 3 representing continuous whole-body myoclonus; 4 representing rearing tonic seizure; and 5 representing tonic–clonic seizure, wild rushing, and jumping. Additionally, the following endpoints were noted: onset of seizures, duration, and percentage survival. Levofloxacin was chosen as a standard due to its broad-spectrum activity and common use in antibacterial assays. Phenytoin was selected as it is a well-validated reference drug in PTZ-induced seizure models.

## 5. Conclusions

This study effectively designed, synthesized, and physiologically tested a novel series of pyrazolone compounds with dual antibacterial and antiepileptic properties. The most promising candidate was discovered as chemical IIa through an integrated computational and experimental method. It revealed considerable in vitro antibacterial activity against clinically relevant strains of *E. coli* and *S. aureus*, as well as potent in vivo anticonvulsant effects in a PTZ-induced seizure model, greatly delaying the seizure onset and assuring 100% survival at a 50 mg/kg dose. Molecular docking simulations revealed a molecular explanation for this dual action, with substantial binding affinities for important bacterial targets (3FYV, 3FRA) and neuronal receptors (4COF, 5L1F) involved in epilepsy. Furthermore, in silico ADMET predictions validated the drug-like properties of the synthesized compounds, consistent with Lipinski’s Rule of Five.

These findings highlight the pyrazolone scaffold as a promising platform to develop multifunctional drugs to combat drug-resistant infections and refractory epilepsy. Finally, this study paves the way for the design of next-generation multifunctional therapeutics. Future studies will include structural optimization to improve potency and selectivity, extensive mechanistic validation using enzyme assays, comprehensive pharmacokinetic and toxicological profiling, and testing against a larger panel of drug-resistant microorganisms. This study lays a solid platform for the development of novel pyrazolone-based treatments against two important global health problems.

## Figures and Tables

**Table 1:** Docking scores of designed ligands.

Ligands	Antiepileptic			Antibacterial		
	4COF	5TP9	5L1F	4URM	3FYV	3FRA
Ia	−9.454	−6.031	−7.106	−6.534	−7.025	−7.037
Ib	−8.842	−6.038	−7.365	−6.564	−7.54	−7.158
Ic	−7.511	−5.818	−6.705	−6.257	−7.104	−7.069
Id	−8.264	−6.057	−7.245	−6.273	−6.807	−6.889
IIa	−7.474	−6.55	−7.409	−6.838	−7.57	−7.499
IIb	−6.476	−5.403	−6.659	−6.342	−7.005	−6.94
IIc	−7.465	−5.594	−6.172	−6.716	−7.088	−7.031
IId	−7.030	−6.126	−6.787	−6.586	−7.043	−6.839
Phenytoin (Std)	−5.13	−5.90	−6.86	-	-	-
Levofloxacin (Std)	-	-	-	−7.24	−6.99	−6.87
Control	−5.33	−5.84	−7.190	−7.82	−6.73	−6.67

Control ligands: I2H (3FRA), XCF (3FYV), XAM (4URM), 7H0 (5TP9), 6ZP (5L1F), and BEN (4COF). Standard drugs: Phenytoin (antiepileptic) and Levofloxacin (antimicrobial). Docking scores are expressed in kcal/mol.

**Table 2:** Drug-likeness properties of synthesized compounds.

Compound Code	Molecular Weight	H-Donar	H-Acceptor	Molar Refractivity	TPSA	Log P	Lipinski Violations
Ia	368.30	1	7	107.57	144.54 Å^2^	1.82	0
Ib	368.30	1	7	107.57	144.54 Å^2^	1.76	0
Ic	368.30	1	7	107.57	144.54 Å^2^	1.44	0
Id	398.32	1	8	114.07	153.77 Å^2^	2.25	1
IIa	245.23	2	4	72.99	95.99 Å^2^	1.17	0
IIb	274.23	1	5	79.79	121.58 Å^2^	1.32	0
IIc	245.23	2	4	72.99	95.99 Å^2^	1.25	0
IId	275.26	2	5	79.48	105.22 Å^2^	1.72	0

**Table 3:** Antibacterial activity of pyrazolone derivatives.

Compound	Conc. (µg/mL)	*E. coli*	*S. aureus*	*P. aeruginosa*
Ia	40	0.00 ± 0.00	0.00 ± 0.00	0.00 ± 0.00
	60	0.00 ± 0.00	0.00 ± 0.00	0.00 ± 0.00
	80	6.00 ± 0.30	0.00 ± 0.00	0.00 ± 0.00
Id	40	0.00 ± 0.00	0.00 ± 0.00	0.00 ± 0.00
	60	0.00 ± 0.00	0.00 ± 0.00	6.00 ± 0.20
	80	0.00 ± 0.00	0.00 ± 0.00	7.00 ± 0.05
IIa	40	7.00 ± 0.18	6.00 ± 0.10	0.00 ± 0.00
	60	7.00 ± 0.26	8.00 ± 0.06	0.00 ± 0.00
	80	9.00 ± 0.10	9.50 ± 0.18	0.00 ± 0.00
Levofloxacin	40	10.00 ± 0.12	12.00 ± 0.17	8.00 ± 0.10
	60	12.00 ± 0.20	13.00 ± 0.10	9.00 ± 0.14
	80	14.00 ± 0.40	15.00 ± 0.10	10.00 ± 0.25

Values are expressed as mean ± SEM based on triplicate measurements. The zone of inhibition: one-way ANOVA Dunnett Comparison, when compared with standard (Levofloxacin).

**Table 4:** Antiepileptic activity via PTZ-induced convulsion method.

Groups	Dose	Mean Onset of Seizures (s)	Mean Duration of Seizures (s)	Seizure Score (Racine)	% Survival
Group I (control)	PTZ (60 mg/kg)	30 ± 0.66	92 ± 0.40	5	50%
Group II	IIa (25 mg/kg)	31 ± 0.38 ^ns^	92 ± 0.44 ^ns^	5	66%
Group III	IIa (37.5 mg/kg)	33 ± 1.28 ^ns^	85 ± 0.61 ^ns^	5	83%
Group IV	IIa (50 mg/kg)	45 ± 0.87 ***	74 ± 0.61 ***	4	100%
Std. Drug (Phenytoin)	20 mg/kg	68 ± 0.74 ***	86 ± 0.61 ***	5	100%

Values expressed as mean ± SEM (“ns” for non-significant; for *p*-values < 0.05, <0.01, and <0.001, respectively). The onset and duration of seizures: one-way ANOVA Dunnett Comparison *** *p* < 0.001, when compared with control.

## Data Availability

The original contributions presented in this study are included in the article/Supplementary Material. Further inquiries can be directed to the corresponding authors.

## Supplementary Materials

The following supporting information can be downloaded at: https://www.mdpi.com/article/10.3390/ph19020193/s1, The FT-IR, 1H NMR, and mass spectrometry spectra of all synthesized compounds are shown in Supplementary Figures S1–S8. Supplementary Figure S1: (A) IR Spectra of compound Ia. (B) NMR Spectra of compound Ia. (C) Mass spectra of compound Ia; Supplementary Figure S2: (A) IR Spectra of compound Ib. (B) NMR Spectra of compound Ib. (C) Mass spectra of compound Ib; Supplementary Figure S3: (A) IR Spectra of compound Ic. (B) NMR Spectra of compound Ic. (C) Mass spectra of compound Ic; Supplementary Figure S4: (A) IR Spectra of compound Id. (B) NMR Spectra of compound Id. (C) Mass spectra of compound Id; Supplementary Figure S5: (A) IR Spectra of compound IIa. (B) NMR Spectra of compound IIa. (C) Mass spectra of compound IIa. Supplementary Figure S6: (A) IR Spectra of compound IIb. (B) NMR Spectra of compound IIb. (C) Mass Spectra of compound IIb; Supplementary Figure S7: (A) IR Spectra of compound IIc. (B) NMR spectra of compound IIc. (C) Mass Spectra of compound IIc; Supplementary Figure S8: (A) IR Spectra of compound IId. (B) NMR Spectra of compound IId. (C) Mass spectra of compound IId.
