# Supplementary material for: Design, Docking, Synthesis, and Biological Evaluation of Pyrazolone Derivatives as Potential Dual-Action Antimicrobial and Antiepileptic Agents
Source: Pharmaceuticals (Basel). 2026 Jan 23;19(2):193. doi: 10.3390/ph19020193 (PMC12943486; doi:10.3390/ph19020193)
Supplement: Supplementary file 1 [file pharmaceuticals-19-00193-s001.zip › pharmaceuticals-4077717-supplementary.pdf]

pharmaceuticals

Article

Design, Docking, Synthesis, and Biological Evaluation of Pyrazolone Derivatives as Potential Dual-Action Antimicrobial and Antiepileptic Agents

Yousef Al-ebini [1], Manojmouli Chandramouli [2], Nagaprashanth Koppuravuri [2,*], Thoppalada Yunus Pasha [2], Mohamed Rahamathulla [3,*], Salwa Eltawaty [4], Kamal Y. Thajudeen [5], Mohammed Muqtader Ahmed [6] and Thippeswamy Boreddy Shivanandappa [7]

1 Department of Cosmetic Science, Faculty of Allied Medical Sciences, Al-Ahliyya Amman University,
Amman 19328, Jordan; y.alebini@ammanu.edu
2 Department of Pharmaceutical Chemistry, Sri Adichunchanagiri College of Pharmacy, Adichunchanagiri University, B. G. Nagara 571448, Karnataka, India; manojmouli@accp.co.in (M.C.)
3 Department of Pharmaceutics, College of Pharmacy, King Khalid University, Abha 62223, Saudi Arabia
4 Department of Biomedical Science, Faculty of Pharmacy, Omar Al-Mukhtar University, Albayda P.O. Box 991, Libya
5 Department of Pharmacognosy, College of Pharmacy, King Khalid University, Abha 61421, Saudi Arabia; kthajudeen@kku.edu.sa
6 Department of Pharmaceutics, College of Pharmacy, Prince Sattam Bin Abdul Aziz University,
Al Kharj 11942, Saudi Arabia; muqtadernano@gmail.com
7 Department of Biomedical Science, College of Pharmacy, Shaqra University, Al-Dawadmi Campus, Dawadmi 11961, Saudi Arabia; drswamy@su.edu.sa
* Correspondence: knp.pharma@accp.co.in (N.K.); rahapharm@gmail.com (M.R.)

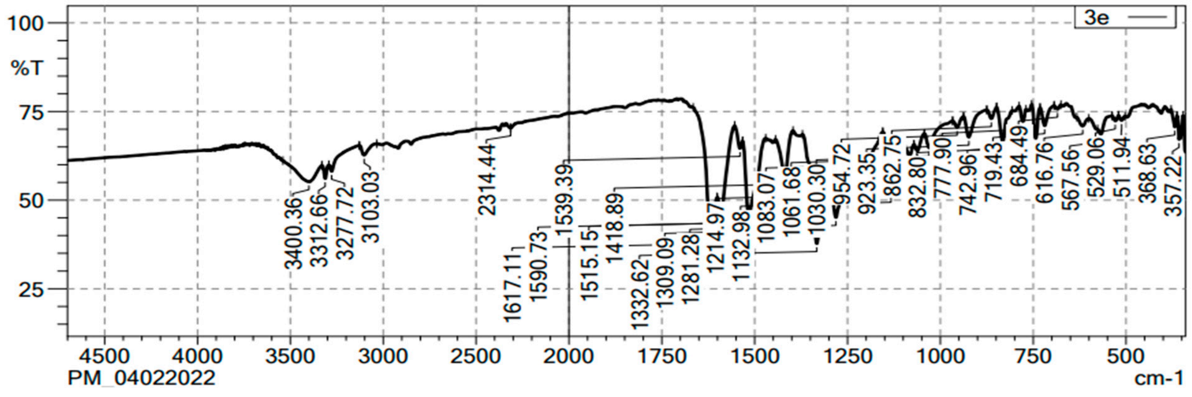

Supplementary Figure S1 A: IR Spectra of compound Ia

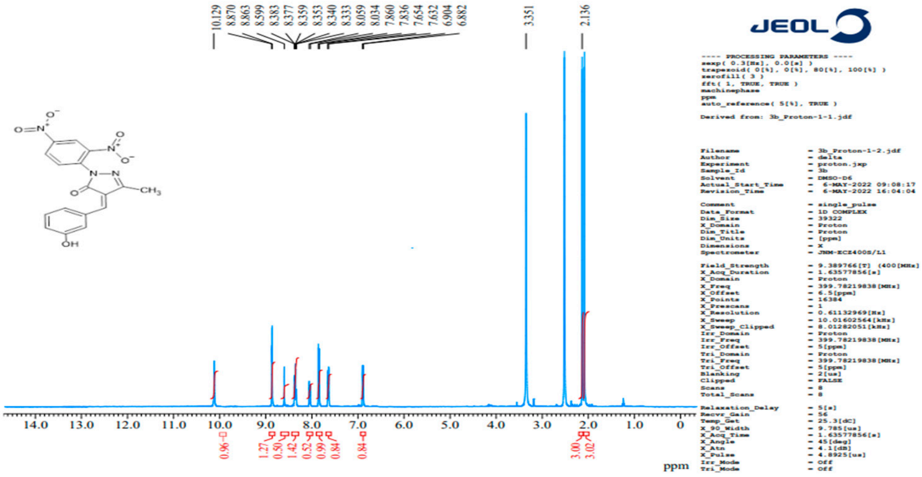

Supplementary Figure S1 B: NMR Spectra of compound Ia

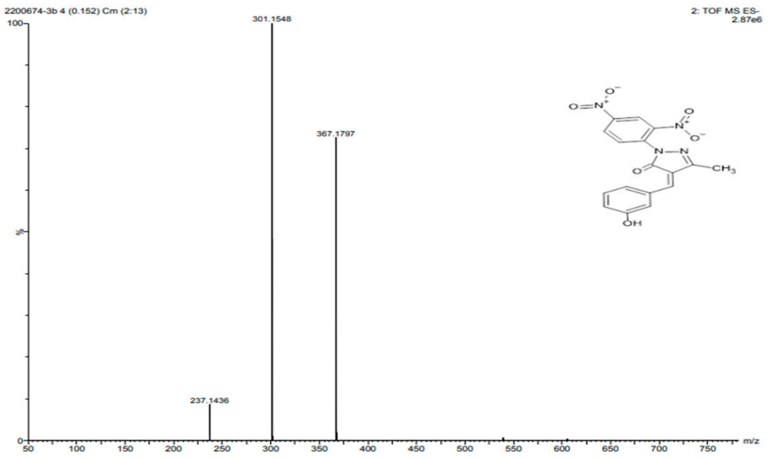

Supplementary Figure S1 C: Mass Spectra of compound Ia

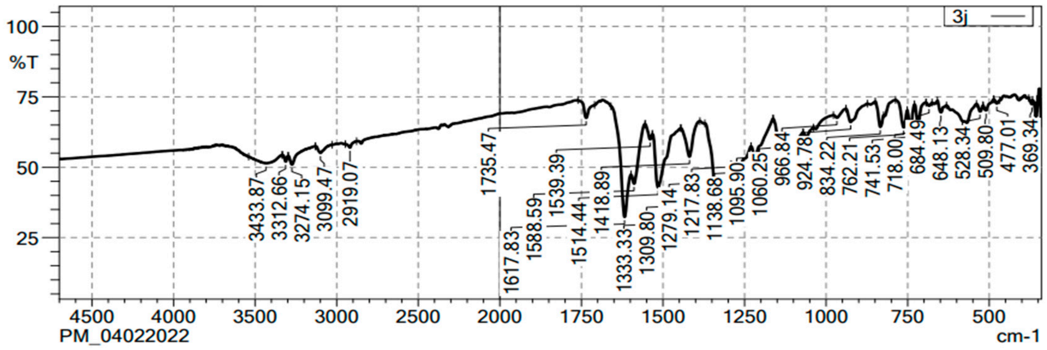

Supplementary Figure S2 A : IR Spectra of compound Ib

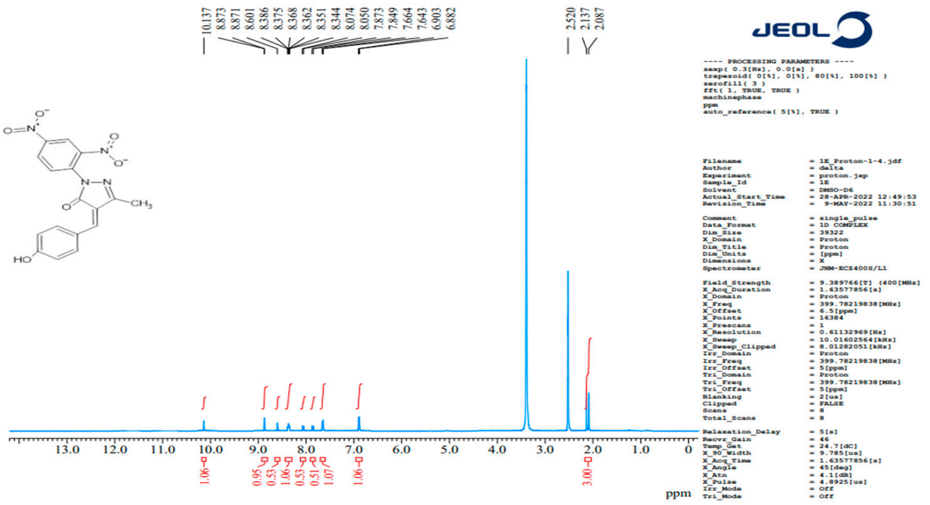

Supplementary Figure S2 B: NMR Spectra of compound Ib

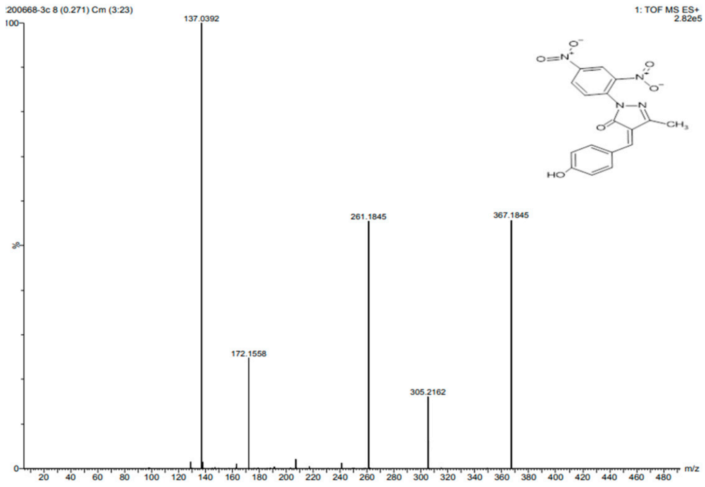

Supplementary Figure S2C: Mass Spectra of compound Ib

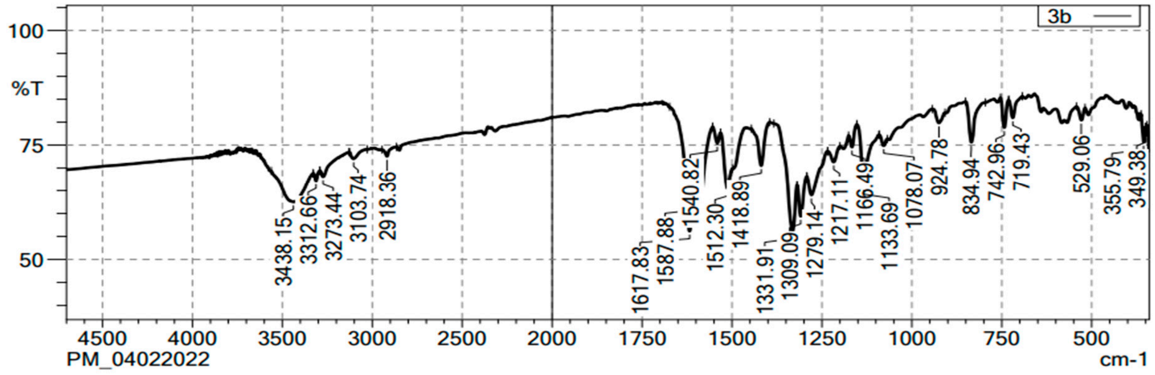

Supplementary Figure S3 A: IR Spectra of compound Ic

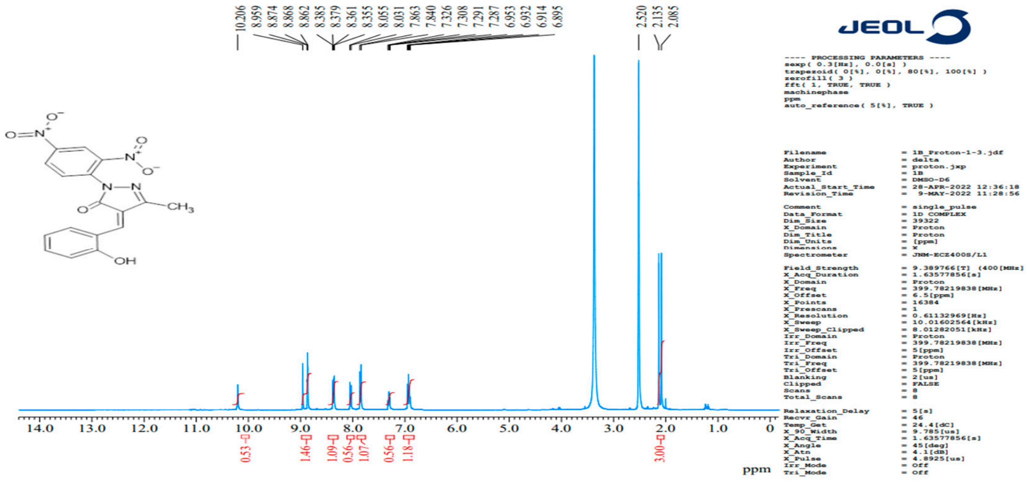

Supplementary Figure S3 B: NMR Spectra of compound Ic

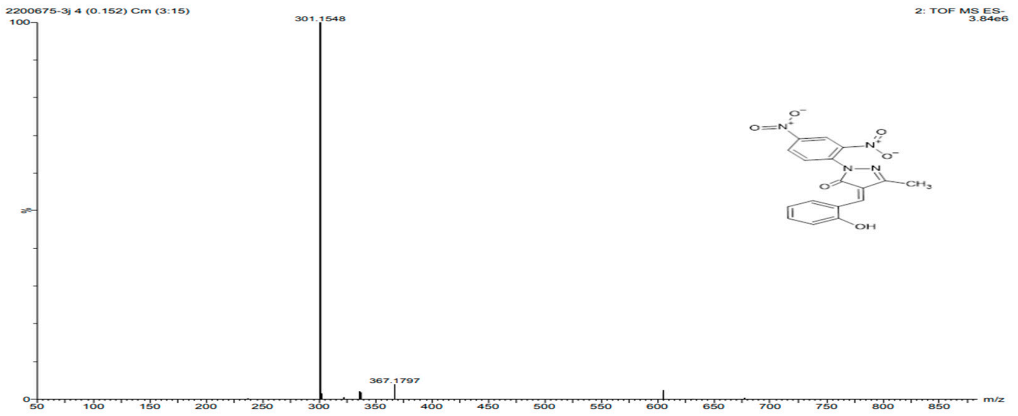

Supplementary Figure S3 C: Mass Spectra of compound Ic

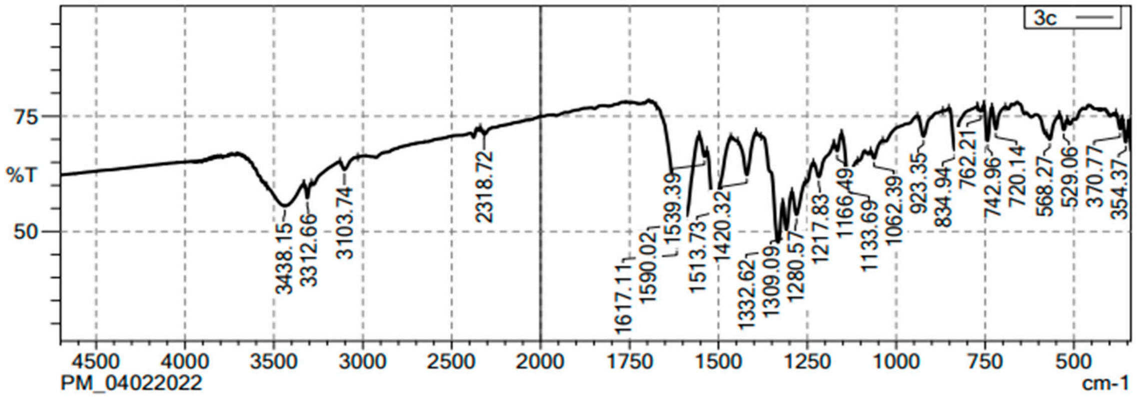

Supplementary Figure S4 A: IR Spectra of compound Id

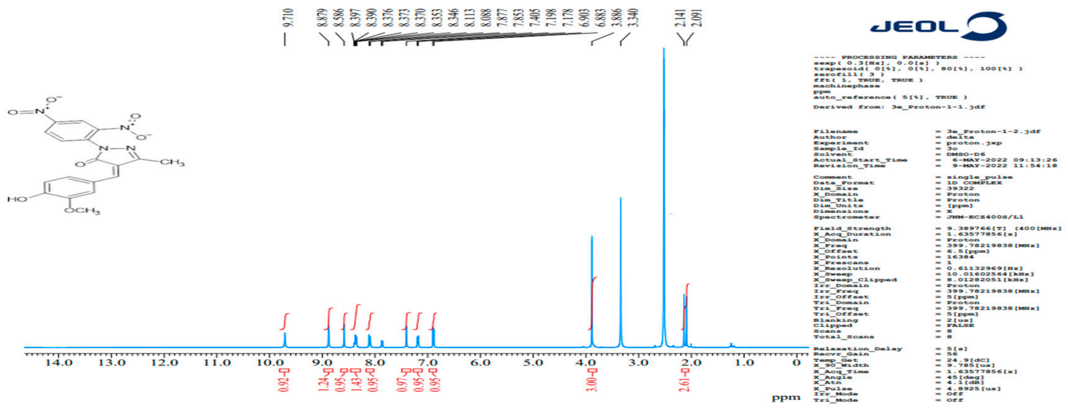

Supplementary Figure S4 B: NMR Spectra of compound Id

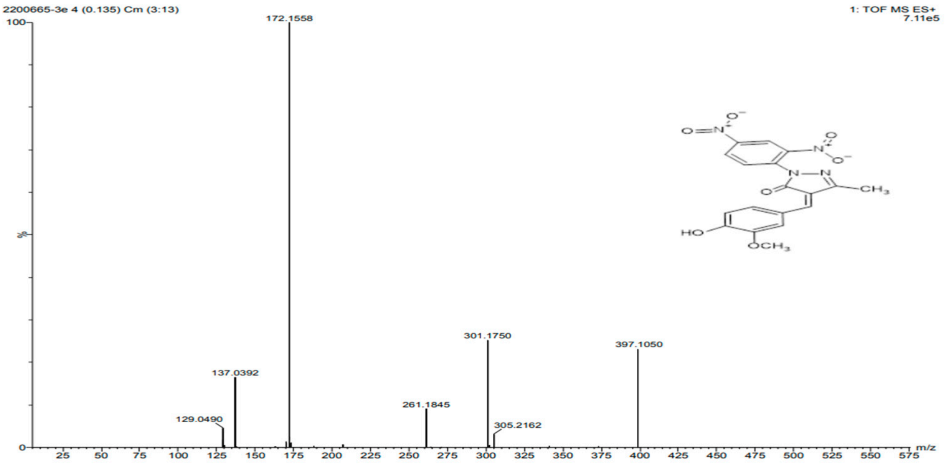

Supplementary Figure S4 C: Mass Spectra of compound Id

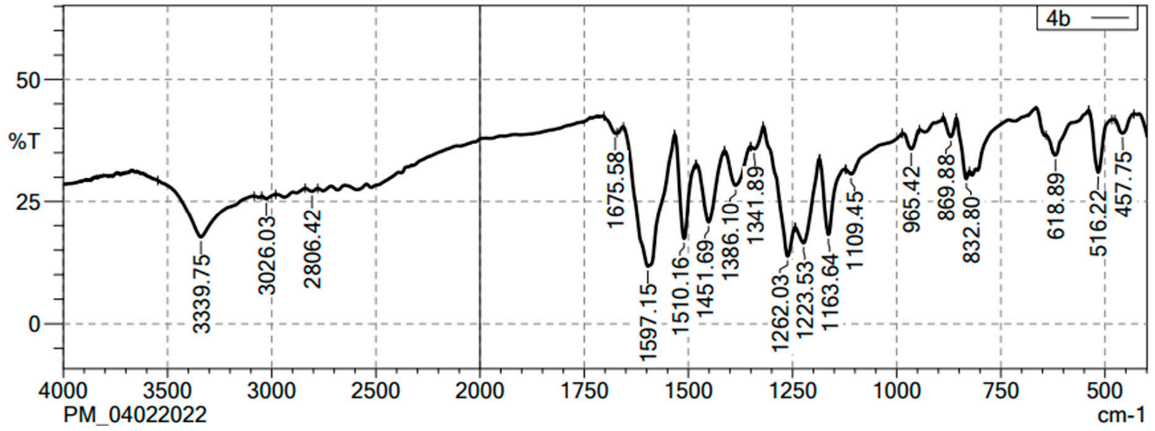

Supplementary Figure S5 A: IR Spectra of compound IIa

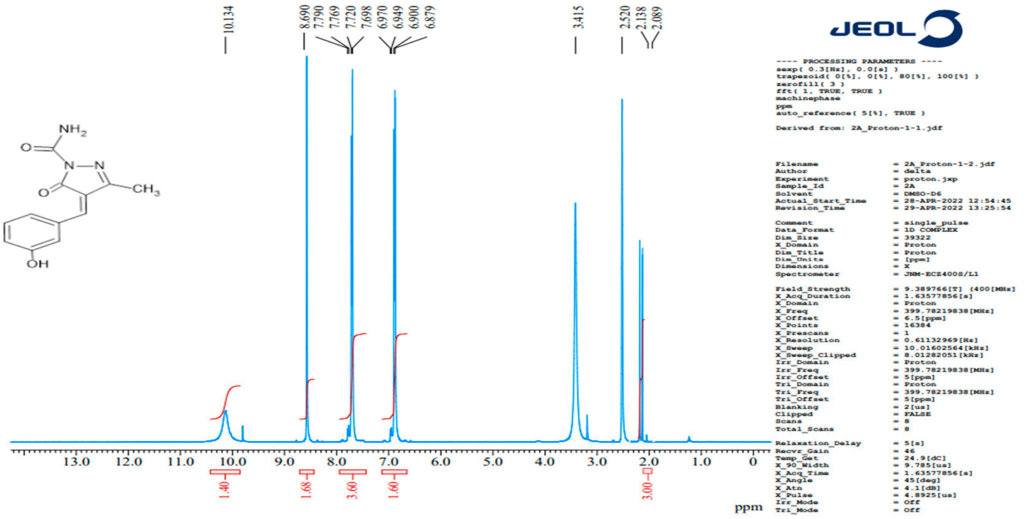

Supplementary Figure S5 B: NMR Spectra of compound IIa

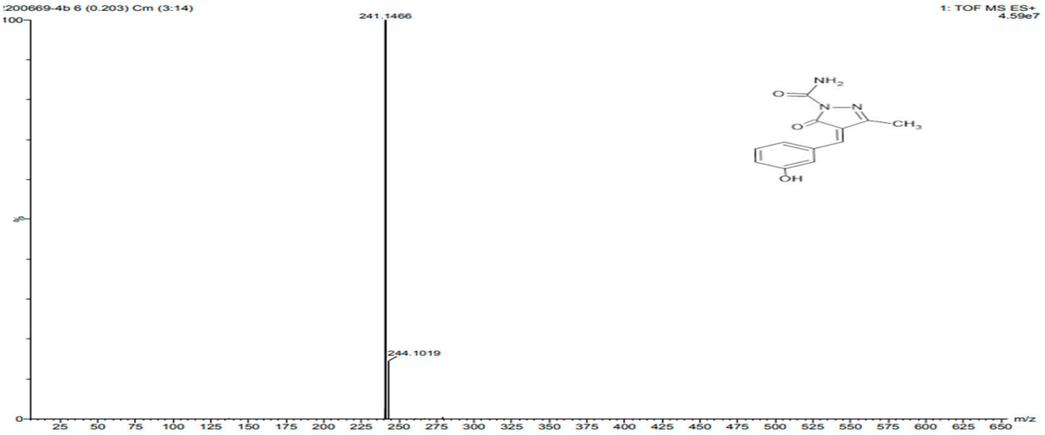

Supplementary Figure S5 C: Mass Spectra of compound IIa

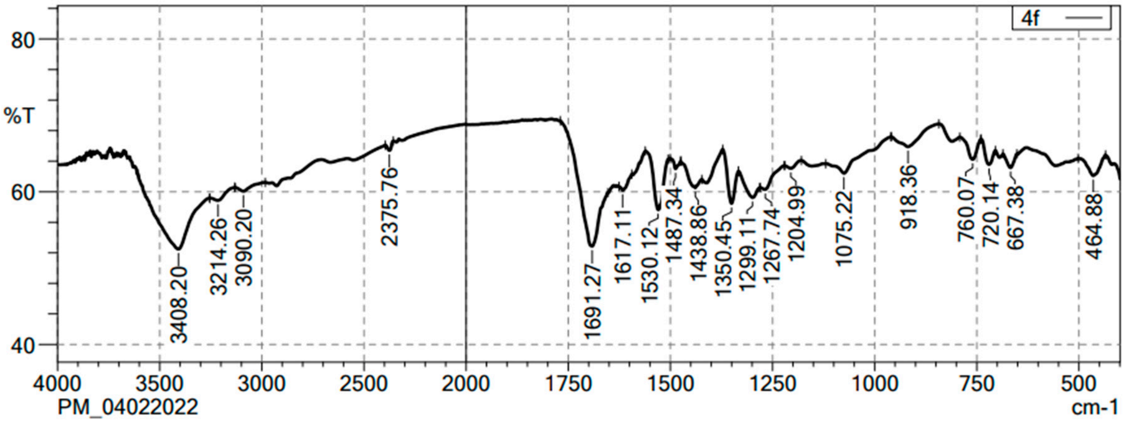

Supplementary Figure S6 A: IR Spectra of compound IIb

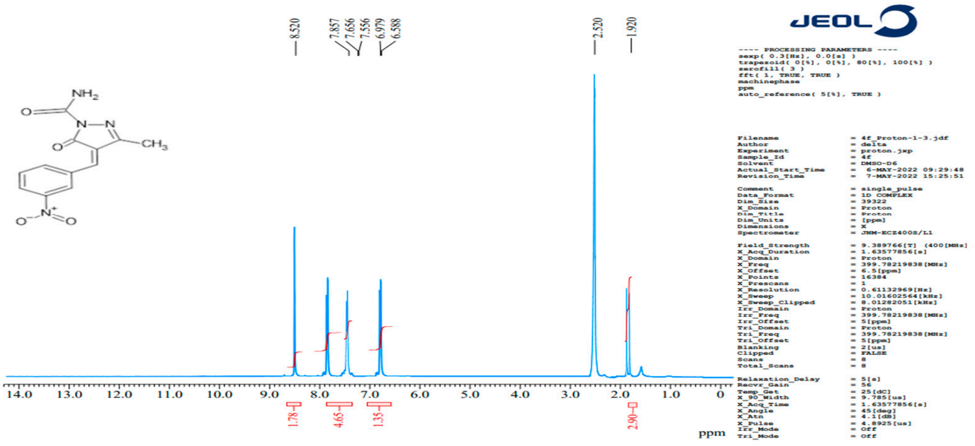

Supplementary Figure S6 B: NMR Spectra of compound IIb

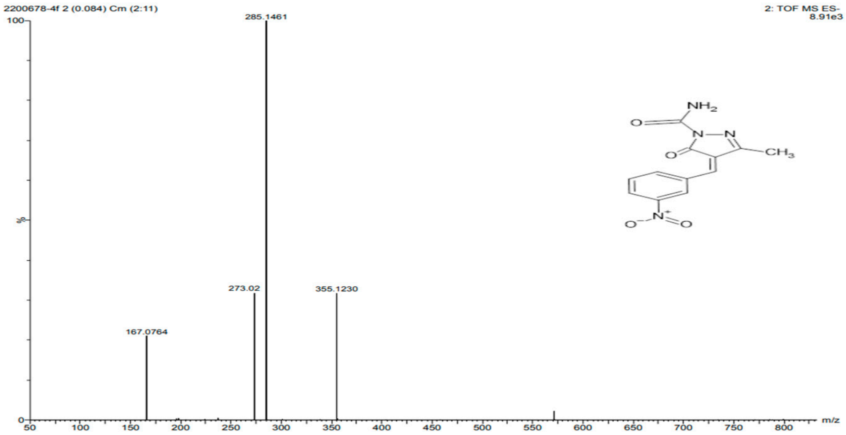

Supplementary Figure S6 C: Mass Spectra of compound IIb

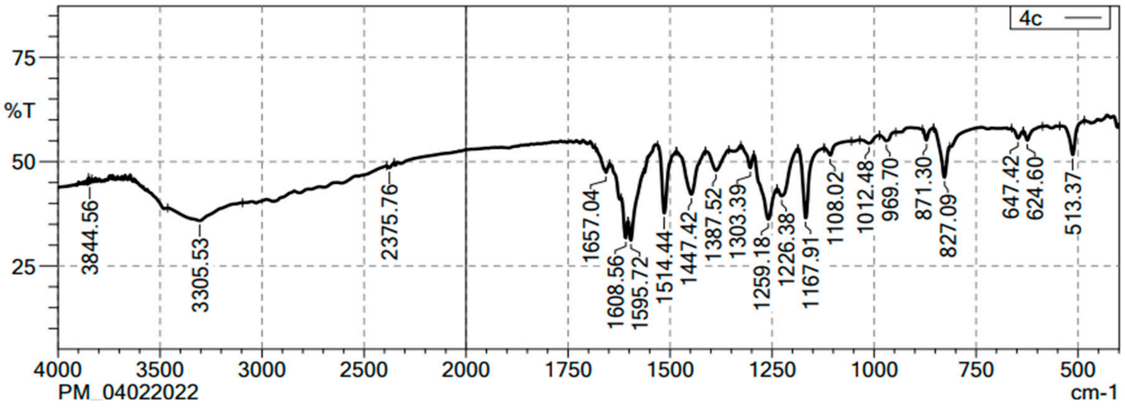

Supplementary Figure S7 A: IR Spectra of compound IIc

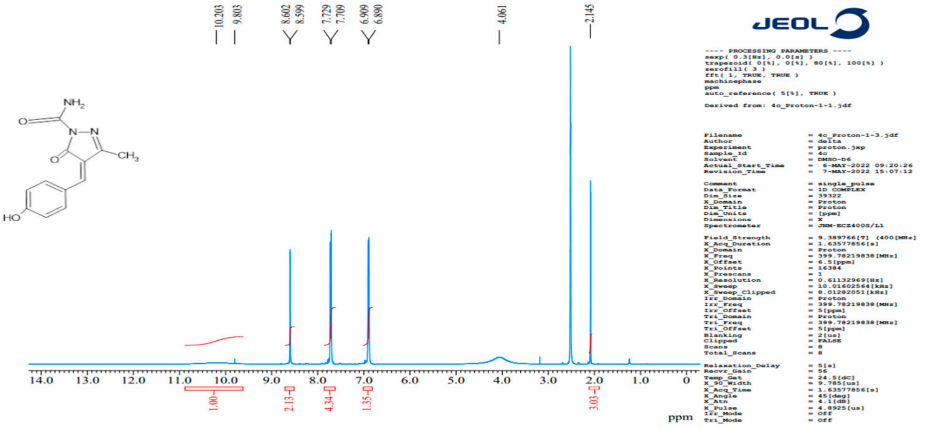

Supplementary Figure S7 B: NMR Spectra of compound IIc

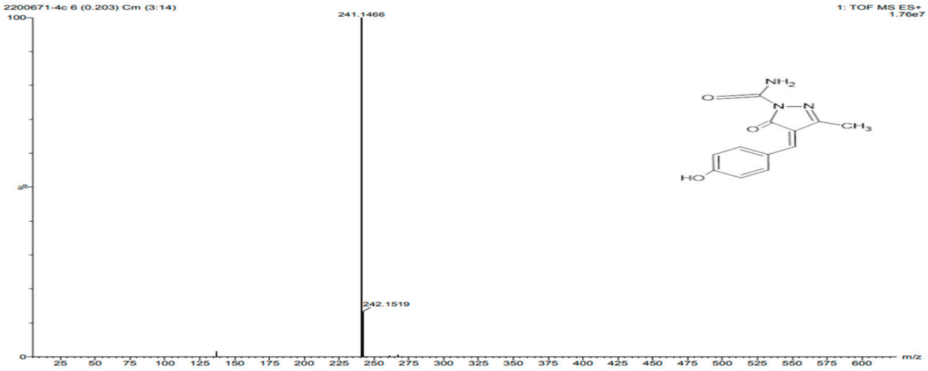

Supplementary Figure S7 C: Mass Spectra of compound IIc

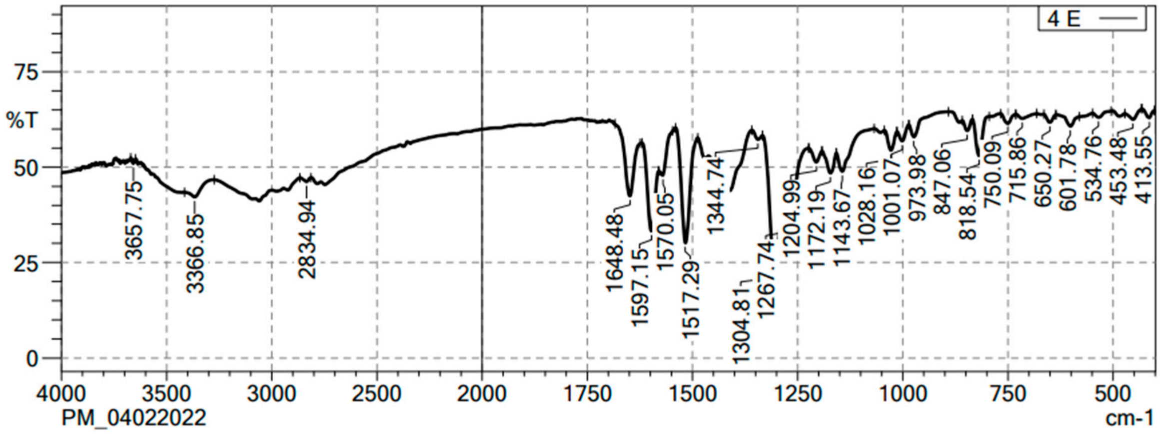

Supplementary Figure S8 A: IR Spectra of compound IId

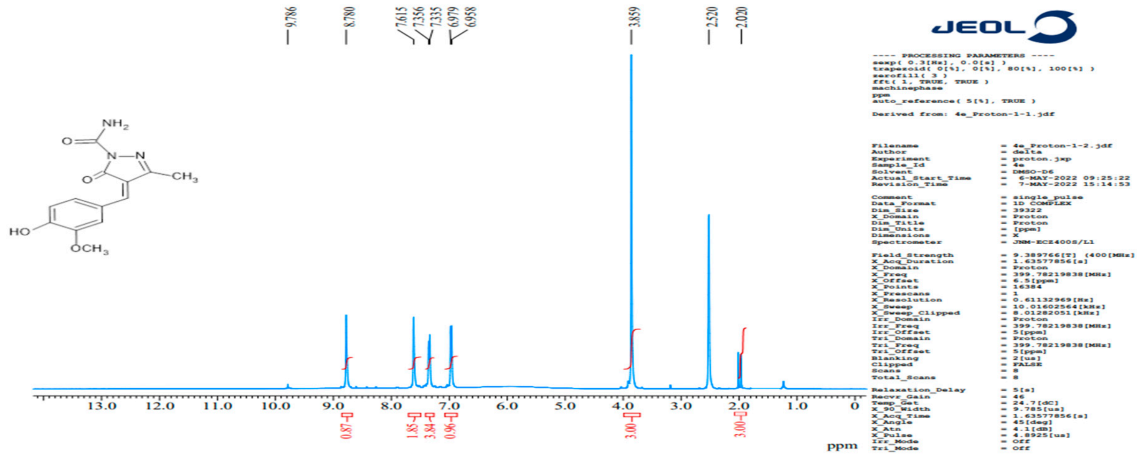

Supplementary Figure S8 B: NMR Spectra of compound IId

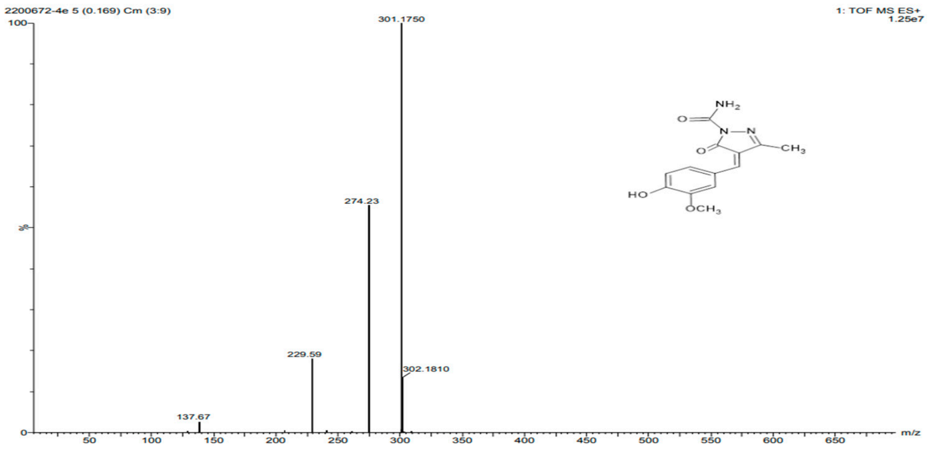

Supplementary Figure S8 C: Mass Spectra of compound IId
